# A high-resolution genome-wide association study of the grain ionome and agronomic traits in rice *Oryza sativa* subsp. *indica*

**DOI:** 10.1038/s41598-021-98573-w

**Published:** 2021-09-28

**Authors:** Suong T. Cu, Nicholas I. Warnock, Julie Pasuquin, Michael Dingkuhn, James Stangoulis

**Affiliations:** 1 College of Science and Engineering, Flinders University, Bedford Park, SA 5042 Australia; 2 Centre for Cancer Biology and ACRF Cancer Genomics Facility, SA Pathology and University of South Australia, Adelaide, SA 5000 Australia; 3 Crop and Environmental Sciences Division, International Rice Research Institute, DAPO Box 7777, Metro Manila, Philippines; 4 UMR AGAP Institut, Cirad, Dept. BIOS, Montpellier, France

**Keywords:** Plant breeding, Plant genetics

## Abstract

This study presents a comprehensive study of the genetic bases controlling variation in the rice ionome employing genome-wide association studies (GWAS) with a diverse panel of *indica* accessions, each genotyped with 5.2 million markers. GWAS was performed for twelve elements including B, Ca, Co, Cu, Fe, K, Mg, Mn, Mo, Na, P, and Zn and four agronomic traits including days to 50% flowering, grain yield, plant height and thousand grain weight. GWAS identified 128 loci associated with the grain elements and 57 associated with the agronomic traits. There were sixteen co-localization regions containing QTL for two or more traits. Fourteen grain element quantitative trait loci were stable across growing environments, which can be strong candidates to be used in marker-assisted selection to improve the concentrations of nutritive elements in rice grain. Potential candidate genes were revealed including *OsNAS3* linked to the locus that controls the variation of Zn and Co concentrations. The effects of starch synthesis and grain filling on multiple grain elements were elucidated through the likely involvement of *OsSUS1* and *OsGSSB1* genes. Overall, our study provides crucial insights into the genetic basis of ionomic variations in rice and will facilitate improvement in breeding for trace mineral content.

## Introduction

Rice (*Oryza sativa* L.) is a major staple food for over half of the world’s population and is a major source of nutrition, although in the form that most consumers eat (white, polished rice), it contains only small amounts of micronutrients^1^. A reliance on rice in the diet, coupled with limited diversity of nutrient-rich foods can lead to malnutrition^2^, with an estimated two billion people suffering from Fe deficiency^3^ and 1.5 billion from Zn deficiency^4^. Fe and Zn are responsible for 2.4% and 1.9%, respectively, of the total global burden of disease^5^.

To combat these deficiencies, various interventions have been used by the nutrition and public health community including supplementation, fortification and in more recent years, biofortification^1^. Both supplementation and fortification can be expensive as they require suitable infrastructure and networks to deliver the nutrient rich product. Biofortification relies on the delivery of Fe- and Zn-dense crops via various strategies, including plant breeding and fertilizer applications^6–8^ and is considered a longer-term sustainable approach where farmers can keep back nutrient-dense seed for subsequent plantings.

Worldwide, the biofortification strategy has led to 300 biofortified varieties being approved for release, in over 40 developing countries^9^ and this includes Zn biofortified rice with moderate levels of Zn, indicating the need for further improvement. The development of Fe-dense rice has not been a target for conventional plant breeding due to insufficient variation for Fe within germplasm, which would not allow for sufficient genetic improvement to have a significant biological effect in humans. The strategy for breeding Fe-dense rice is through a transgenic route, with the overexpression of nicotianamine synthase genes showing significant promise in delivering Fe to deficient communities^9,10^.

Conventional breeding for Zn-dense rice is challenging. While sufficient variation for Zn exists in the germplasm and this allows for a breeding strategy to be undertaken, there is moderate heritability^11^ and therefore stability of the Zn-dense trait across environments is a major challenge. Understanding of the various environmental factors and genes impacting on the scavenging of Zn from the root rhizosphere and the short/long distance transport routes to the developing caryopsis has come a long way^12–14^ but there remain major gaps in our understanding and this confounds selection of stable parents. The introduction of stable genetic markers would be advantageous to accelerate development of Zn-dense rice.

GWAS is a powerful tool to study the molecular basis for phenotypic diversity in rice. Compared with conventional biparental population linkage mapping, GWAS has two outstanding advantages: (i) the rice varieties/accessions used in GWAS panels often contain much more genetic diversity and (ii) GWAS can take full advantage of numerous ancient recombination events resulting in higher mapping resolution^15^. Over the last decades, studies using GWAS platforms have successfully dissected the genetic bases of several complex traits in major crops, such as flowering time and yield-related traits^16–18^. There have also been studies investigating the genetics controlling the element accumulation in rice grain resulting in the identification of significantly associated loci and putative casual genes^11,19–21^. Examples of the identified genes include the *OsHMA3* transporter gene controlling the translocation of Cd from the roots to the shoots^22^ and the molybdate transporter *OsMOT1* gene controlling molybdenum concentration in both straw and grain^23^.

Many factors can affect the efficacy of GWAS such as population structure, sample size and marker density. The rice diversity panel used in this study consisted of 233 *Oryza sativa* subsp. *indica* genotypes. This panel was developed at the International Rice Research Institute (IRRI), Philippines for the Phenomics of Rice Adaptation and Yield potential (PRAY) project as a part of the Global Rice Phenotyping Network (http://ricephenonetwork.IR.org/diversity‐panels/pray‐diversity‐panel). The panel represented the diversity within the *indica* sub-species covering improved and traditional varieties across subtropical and tropical regions around the world^24^. Previously, this panel was used in GWAS studies for various traits including grain quality, panicle architecture, root plasticity, grain yield and yield-related traits^24–28^. This panel was initially genotyped with 700,000 SNPs^29^ and in the latest restatement, 5.2 million biallelic SNPs have been imputed on this panel by comparing the 700,000 SNPs with whole-genome sequence data of the 3000 sequenced rice cultivars^30^. The imputed high-density SNP set aimed to increase the signal strength of the marker–trait associations (MTA) and improve the mapping resolution.

In this study, we investigated the performance of twelve elements in brown rice grain of the diversity panel grown in four environments. GWAS were carried out to identify significant association loci that were stably expressed in the multiple environments. Subsequently, multiple potential causal candidate genes were identified and a genetic mechanism underlying the correlations among different trace minerals were proposed. Favourable alleles and candidate genes for improved micronutrient nutrition, especially for zinc, were identified that could be used in rice biofortification programs.

## Results

### Genotypic markers and population structure

The number of independent markers in the *indica* accessions was estimated to be 6591. Using this figure in a Bonferroni correction gave a corrected significance threshold of p < 7.59 × 10^–6^ (= 0.05/6591) or a − log_10_(p) > 5.12 for use in declaration of significant MTA.

**Figure 1:**
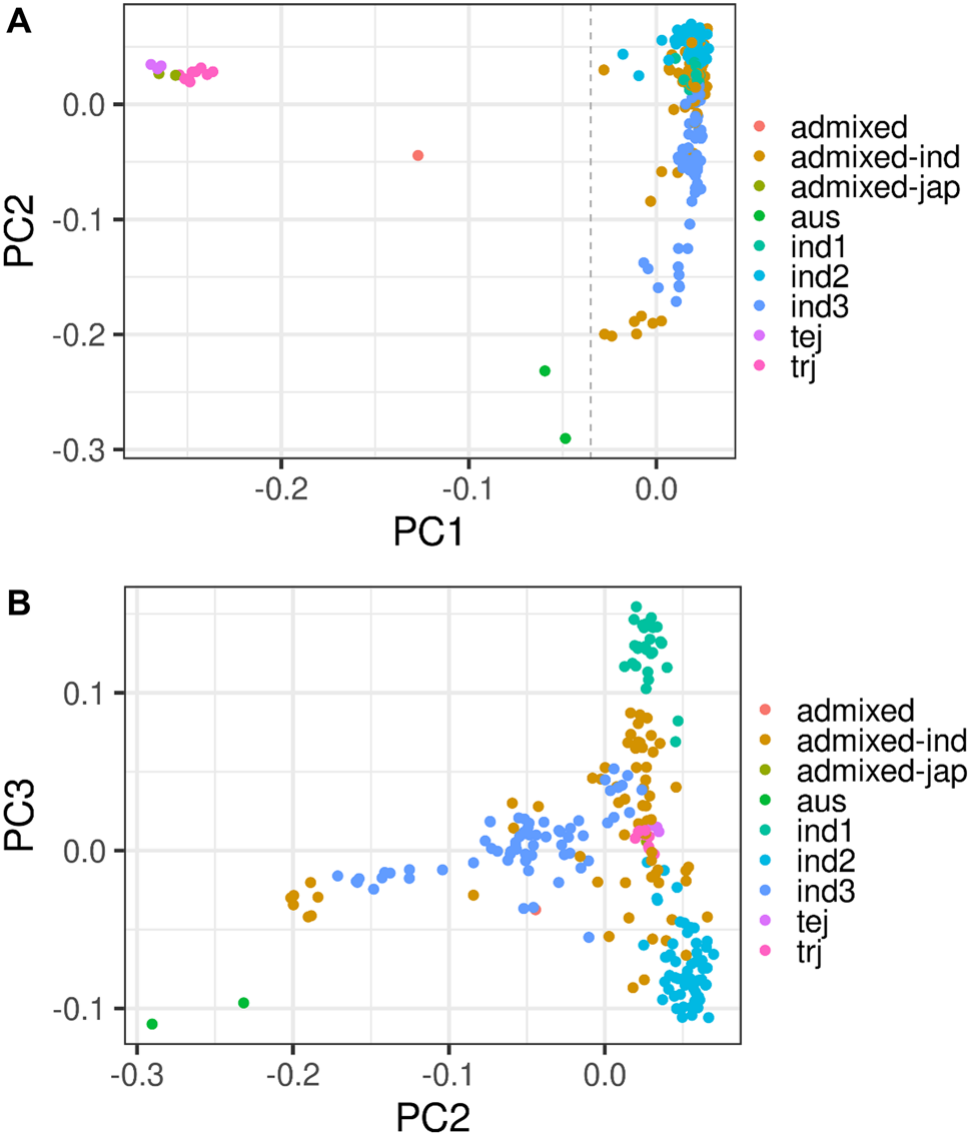
Population structure in PRAY Indica panel indicated by principal component analysis. Principal component analysis was performed in genotypic data in Plink, which separate different subpopulations identified by Wang, et al.^30^. PC1 vs PC2 (**A**) separates Indica accessions from Japonica; PC2 v PC3 (**B**) separates Indica subpopulations.

Population structure within the panel was examined using principal component analysis. The first PC (PC1) was sufficient to discriminate *indica* from *aus* and *japonica* accessions (Fig. 1). PC2 and PC3 further separated *indica* accessions into three distinct groups. Wang et al*.*^30^ termed the three *indica* sub-populations IND1, IND2 and IND3 with their origins mapped broadly to China (1); Indonesia and the Philippines (2); and India and Pakistan (3). The principal component analysis identified 17 non-*indica* accessions in the population, consistent with allocations made by Wang et al*.*^30^. Non-*indica* accessions were removed from this study. The first 2 PCs were used as covariates for association analyses due to their representation of real *indica* sub-populations.

### Phenotypic variation and trait heritability

**Figure 2:**
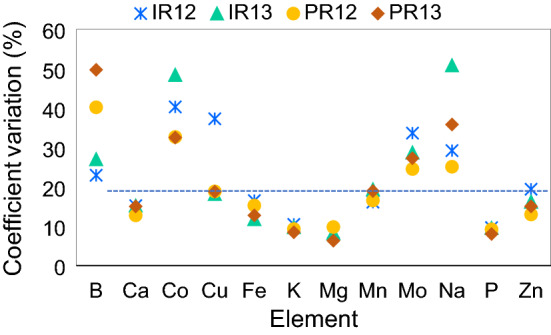
The coefficient of variation (%) of 12 elements in the grain the rice panel grown at four environments (IR12: IRRI 2012, IR13: IRRI 2013, PR12: PhilRice 2012, PR13: PhilRice 2013).

The concentrations of 12 element (B, Ca, Co, Cu, Fe, K, Mg, Mn, Mo, Na, P and Zn) of the brown rice grain from the panel and their broad-sense heritability values are presented in Table 1. The scale of ionomic variation depends on both the element and the growing environment (Table 1, Fig. 2). Amongst the 12 elements, the lowest variation in concentrations were found with the three macronutrients: K, Mg and P (coefficient variation (CV) ranging from 6 to 10%), followed by Ca, Fe, Mn and Zn (CV ranging from 12 to 19%). The largest variations were found with the five elements: B, Co, Cu, Mo and Na (CV ranging from 19 to 51%). The agronomic traits including days to 50% flowering (DF), grain yield (GY), plant height (PH) and thousand grain weight (TGW) were also included in Table 1. Amongst these traits, GY (CV ranging from 25 to 50%) varied considerably more than the other three traits: PH, DF and TGW (CV ranging from 12 to 22%).

**Table 1:** Descriptive statistics of agronomic and grain element traits in the PRAY panel grown in four environments (IR12: IRRI 2012, IR13: IRRI 2013, PR12: PhilRice 2012, PR13: PhilRice 2013). Data represents the mean ± standard deviation. Different letters indicate statistically significant differences between growing environments at P < 0.05 (ANOVA, one-way, Bonferroni pairwise test). All element concentrations were expressed as mg kg^−1^; DF, days to 50% flowering; PH, plant height (cm); GY, grain yield (kg ha^−1^); TGW: thousand grain weight (mg); H^2^, broad-sense heritability; –, data not available.

Trait	IR12	IR13	PR12	PR13	H^2^
B	6.4 ± 1.5^b^	13.1 ± 3.5^a^	2.5 ± 1.0^d^	4.2 ± 2.1^c^	0.26
Ca	96.8 ± 14.7^b^	106.1 ± 16.2^a^	99.3 ± 12.7^b^	104.4 ± 15.7^a^	0.90
Co	0.043 ± 0.017^c^	0.052 ± 0.025^a^	0.048 ± 0.016^b^	0.055 ± 0.18^a^	0.79
Cu	4.6 ± 1.7^b^	5.9 ± 1.1^a^	3.3 ± 0.6^c^	3.2 ± 0.6^c^	0.55
Fe	11.9 ± 2.0^b^	12.6 ± 1.5^a^	10.6 ± 1.6^c^	11.7 ± 1.5^b^	0.76
K	3287 ± 343.7^b^	3521 ± 343^a^	2862 ± 266.2^c^	3471 ± 294^a^	0.82
Mg	1455 ± 123.8^c^	1485 ± 122.4^b^	1268 ± 124.4^d^	1516 ± 96.1^a^	0.70
Mn	28.3 ± 4.6^b^	33.3 ± 6.4^a^	23.7 ± 3.9^d^	25.1 ± 4.7^c^	0.83
Mo	1.03 ± 0.35^b^	1.56 ± 0.45^a^	0.40 ± 0.098^c^	0.36 ± 0.099^c^	0.73
Na	13.8 ± 4.0^b^	19.8 ± 10.1^a^	10.3 ± 2.6^c^	10.0 ± 3.6^c^	0.47
P	3882 ± 374^b^	4014 ± 383^a^	3445 ± 317^c^	3994 ± 320^a^	0.79
Zn	25.2 ± 4.9^a^	23.3 ± 3.8^b^	21.0 ± 2.7^d^	22.1 ± 3.3^c^	0.85
DF	93.3 ± 17.5^a^	77.6 ± 10.7^c^	74.8 ± 11.3^c^	81.0 ± 9.7^b^	0.76
GY	274.0 ± 136.6^d^	417.4 ± 141.8^c^	1003 ± 281.3^a^	616.5 ± 152.8^b^	0.38
PH	151.7 ± 34.1^a^	139.5 ± 29.6^b^	129.4 ± 18.9^c^	131.9 ± 24.9^c^	0.89
TGW	–	17.0 ± 2.5^b^	–	17.7 ± 2.5^a^	–

The growing environment had significant effects on all measured traits (one-way ANOVA, p < 0.05) (Table 1). GY was significantly different between all four environments. The highest GY was observed at PR12, approximately 1.6-, 2.4- and 3.7-fold higher than those at PR13, IR13 and IR12, respectively. The panel grown in the wet season (IR12) had significantly taller plants and longer DF than those grown in the dry seasons.

The highest yielding environment (PR12) had the lowest concentrations of 11 elements; all except for Co. On the contrary, the third highest yielding environment (IR13) had the highest concentrations of 10 elements (all except for Mg and Zn). The grain Zn concentration had the exact reverse ranking of the GY, with the highest concentration was from IR12, followed by IR13, PR13 and PR12.

The broad-sense heritability values for all the traits ranged from 26 to 90%. High heritabilities (> 70%) were observed for the nine grain elements (Ca, Co, Fe, K, Mg, Mn, Mo, P and Zn) and the two agronomic traits (DF and PH). The three grain elements (B, Cu and Na) and GY were estimated to have low to moderate heritabilities (26–55%).

### Correlations between traits and environments

Significant correlations between all four environments were observed for 10 elements (all except for B and Cu) and PH (p < 0.01, r: 0.25–0.93) (Supplementary Table S1). The group that had consistently high correlation coefficients (r > 0.5) between all environments included Ca, Mo, Mn, K and PH. For GY, significant correlations were found for the panel grown at the same site; between IR12 and IR13 (r: 0.36) and between PR12 and PR13 (r: 0.41). For DF, high correlations (r: 0.85–0.87) were found between the three dry seasons (IR13, PR12 and PR13).

Trait-wise correlation analysis within each environment reveals that the concentrations of the five elements; Zn, Fe, K, Mg, P were significantly correlated with each other in all environments (p < 0.05) (Supplementary Table S2). Of the five elements, P concentration consistently had the highest correlation coefficients with the other four elements in all environments: Zn and P (r: 0.45–0.57), Fe and P (0.41–0.59), K and P (0.60–0.74) and Mg and P (r: 0.73–0.89). These five elements also had strong correlations to the other elements including Cu (3/4 environments), Mn (3/4), Mo and Co (2/4).

Significant correlations were also observed between element concentrations and the other traits within each environment (Supplementary Table S2). Specifically, GY was negatively correlated with the grain Zn, Fe, K, Mg and P concentrations in all environments, with the Cu, Mn, Mo and Na concentrations in three and with B, Ca and Co in two environments. PH had positive correlations with the Ca, Co, K, Mg, Na, P and Zn concentrations in two or more environments and negative correlations to Cu and Mo concentrations in three environments. DF had negative correlations six elements: namely Ca, Co, Cu, Fe, Mg, P in two or more environments.

The correlations between developmental and agronomic traits differed markedly between the growing environments. GY was positively correlated to DF and PH in two seasons (PR12 and PR13) and negative correlated with PH in one (IR13). PH and DF had only one significant correlation in the wet growing season IR12 (r:0.58).

### Detection of stable and environment-specific QTL

GWAS was performed separately for each environment and identified 128 QTL for grain element concentrations and 57 QTL for agronomic traits (Supplementary Tables S3 and S4).

#### QTL associated with grain elements

The QTL identified for grain elements were distributed on all chromosomes (Supplementary Table S3). The highest number of QTL was detected for Mo (22 QTL), followed by B, Co, Fe, K, Mn, Na and Zn (10–19 QTL, each) and Ca, Cu, Mg and P (3–5 QTL, each). Environment-wise, the highest numbers of QTL were detected for the two dry growing seasons IR13 and PR13 (45 and 43 QTL, respectively), followed by PR12 (35 QTL) and IR12 (25 QTL).

Of the grain element QTL, 14 were consistently identified in two or more environments (Table 2). There was one QTL that was common in all four environments; namely *qZn7.2*, three QTL stable in three environments (*qCo7.1*, *qK6.1* and *qZn7.2*) and ten QTL common in two environments (*qB7.1, qCa3.1, qCa12.2, qCo8.1, qFe1.2, qMo3.3, qMo8.1, qMo10.2, qNa1.2, qNa11.5*). Elements having multiple stable QTL were Ca, Co, K, Mo, Na, Zn while Cu, Mg, Mn and P had no stable QTL.

**Table 2:** Summary of the stable QTL detected in 2, 3 and 4 environments (highlighted in bold, italics and bolditalics, respectively. *Env* environment (1 = IRRI 2012, 2 = IRRI 2013, 3 = PhilRice2012, 4 = PhilRice2013), *Chr* chromosome, *Start/End* physical Mb position of the linkage block, *Add* Effect: estimated additive effect, *PVE* phenotypic variation explained by the QTL (%), *High all Freq* Frequency of the higher value allele.

Trait	QTL	Env	Chr	Start Mb	End Mb	− log_10_P	PVE%	Add. effect	High all Freq
B	**qB7.1**	1	7	6.06	6.49	8.8	9.7	2.44	0.12
	**qB7.1**	2	7	6.06	6.49	8.9	11.5	4.03	0.16
Ca	**qCa3.1**	1	3	16.66	16.92	6.0	9.5	15.12	0.56
	**qCa3.1**	4	3	16.80	16.90	5.7	5.1	13.52	0.41
	**qCa12.2**	3	11	27.83	27.83	5.2	4.7	6.03	0.66
	**qCa12.2**	4	11	27.83	27.83	5.2	3.6	7.89	0.68
Co	*qCo7.1*	2	7	29.23	29.37	5.5	4.5	0.02	0.14
	*qCo7.1*	3	7	29.26	29.35	7.2	6.2	0.03	0.14
	*qCo7.1*	4	7	29.27	29.34	5.4	5.1	0.02	0.14
	**qCo8.1**	3	8	3.55	3.56	5.5	12.4	0.00	0.09
	**qCo8.1**	4	8	3.55	3.57	6.2	15.7	0.00	0.90
Fe	**qFe1.2**	2	1	2.53	2.64	5.9	6.7	0.41	0.60
	**qFe1.2**	4	1	2.56	2.66	5.6	7.0	0.44	0.82
K	*qK6.1*	2	6	1.59	1.83	7.8	6.2	255.44	0.13
	*qK6.1*	3	6	1.70	1.81	5.5	5.0	261.83	0.13
	*qK6.1*	4	6	1.70	1.83	6.0	4.5	233.69	0.13
Mo	**qMo3.3**	1	3	26.70	26.83	6.4	7.2	0.67	0.05
	**qMo3.3**	3	3	26.76	26.78	6.0	6.3	0.17	0.06
	**qMo8.1**	3	8	0.00	0.25	9.2	10.1	0.12	0.85
	**qMo8.1**	4	8	0.00	0.33	8.0	9.9	0.07	0.38
	**qMo10.2**	2	10	5.17	5.28	5.4	4.4	0.10	0.09
	**qMo10.2**	3	10	5.17	5.36	7.1	7.5	0.59	0.09
Na	**qNa1.2**	1	1	11.01	11.54	8.9	9.7	10.28	0.27
	**qNa1.2**	2	1	11.46	11.52	6.3	5.2	2.75	0.77
	**qNa11.5**	2	11	27.37	27.48	6.2	5.4	5.54	0.05
	**qNa11.5**	4	11	27.68	27.82	5.2	4.8	10.73	0.16
Zn	***qZn7.2***	**1**	**7**	**29.26**	**29.43**	6.3	5.0	4.88	0.11
	***qZn7.2***	**2**	**7**	**29.26**	**29.33**	6.4	5.1	3.87	0.16
	***qZn7.2***	**3**	**7**	**29.26**	**29.42**	6.9	8.0	2.94	0.19
	***qZn7.2***	**4**	**7**	**29.26**	**29.41**	6.1	5.2	7.34	0.06
	*qZn7.3*	1	7	29.42	29.67	6.5	5.4	6.38	0.08
	*qZn7.3*	2	7	29.47	29.67	5.5	4.3	4.04	0.12
	*qZn7.3*	3	7	29.52	29.67	5.4	5.0	4.00	0.07

The proportions of phenotypic variation explained (PVE) by these QTL ranged from 3.6 to 15.7% (Supplementary Table S3). The QTL having the largest proportion of PVE in each environment were: *qB4.3*, *qMn9.1* and *qCu1.1* (11.8%, 10.5% and 10.2%, respectively) in IR12; *qB7.1, qCu4.1* and *qFe7.2* (11.5%, 10.4% and 10.1%, respectively) in IR13; *qCo8.1, qB2.1* and *qMo8.1* (15.7%, 10.9% and 9.9%, respectively) in PR12; *qCo8.1, qMg3.1* and *qMo8.1* (12.4%, 10.3% and 10.1%, respectively) in PR13.

#### Combined effect of the QTL for grain Zn concentration

**Figure 3:**
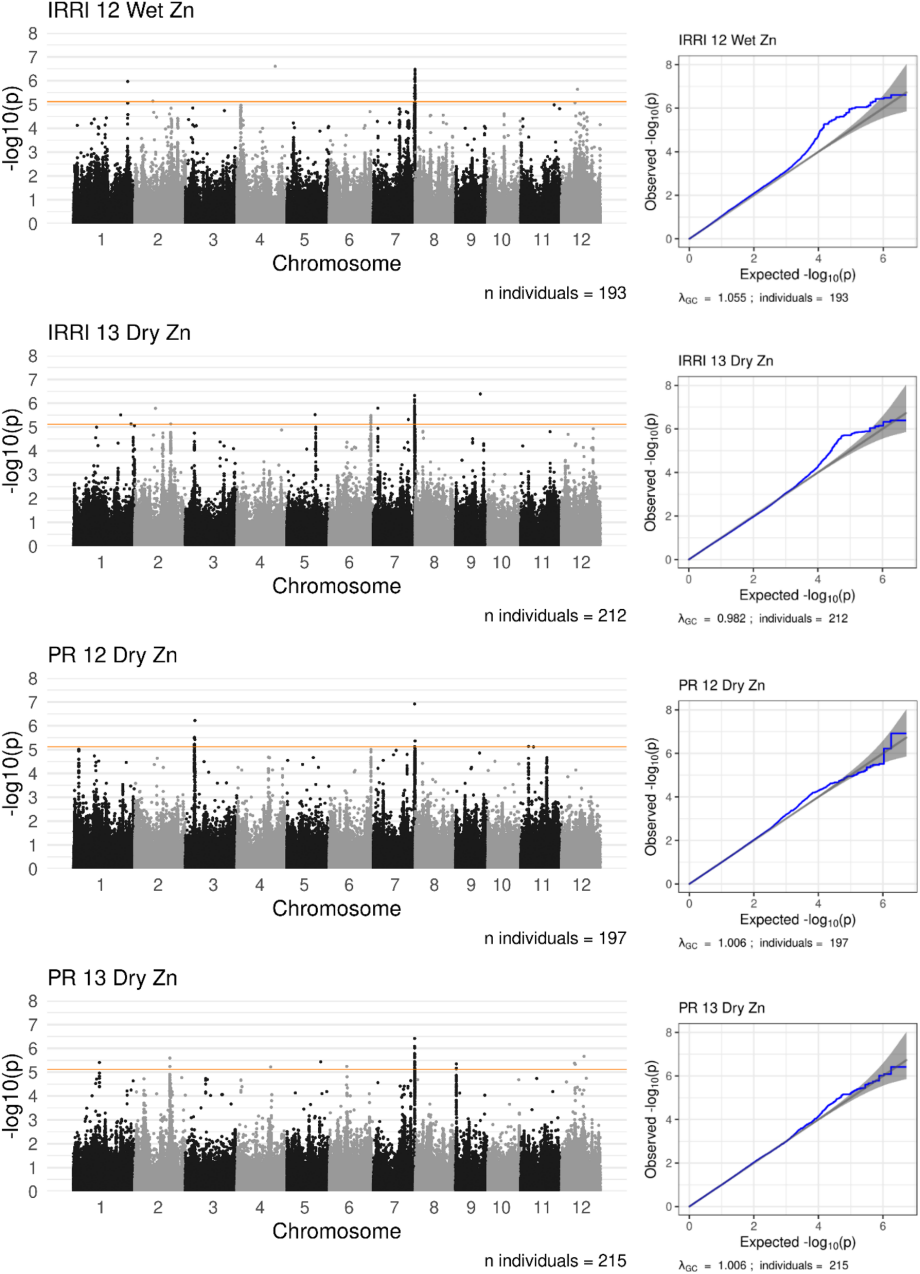
Manhattan plots (left) and QQ plots (right) showing results of association analysis for zinc concentration in 2012 IRRI wet season (IRRI 12 Wet Zn), 2013 IRRI dry season (IRRI 13 Dry Zn), 2012 PhilRice dry season (PR 12 Dry Zn) and 2013 PhilRice dry season (PR 12 Dry Zn). The orange line indicates a significance threshold of − log_10_(p) > 5.12.

A total 13 QTL identified for Zn concentration in four environments (Fig. 3, Supplementary Table S3). The combined QTL effects explained for approximately 19.7–32.1% of the variation in Zn concentration in each environment. The highest additive effects in each environment were 7.3 mg kg^−1^ (*qZn7.2* in IR12), 5.4 mg kg^−1^ (*qZn6.1* in IR13), 4.0 mg kg^−1^ (*qZn7.3* in PR12) and 4.3 mg kg^−1^ (*qZn1.1* in PR13). In all of those cases, high Zn was associated with the minor alleles (6–8% allele frequency). Two of the QTL (*qZn7.2* and *qZn7.3*) were stable across three and four growing environments, respectively.

**Figure 4:**
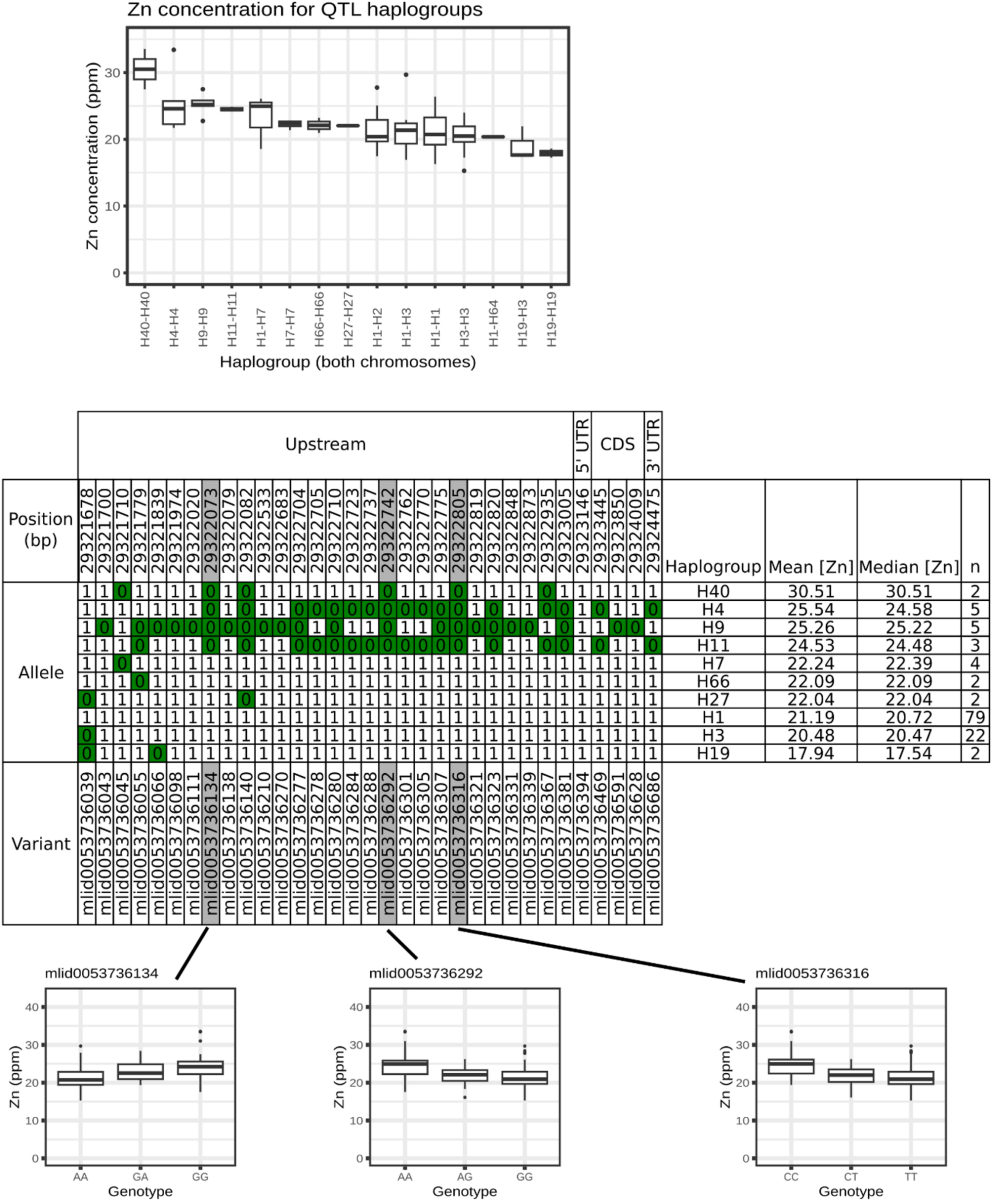
Haplotype analysis of *OsNAS3* (a candidate gene under four- environment Zn QTL *qZn7.2*, including 1.5 kb upstream from start codon. Top: boxplot of grain zinc concentration associated with each haplotype. Middle: SNP analysis of the same region for homozygous haplotypes, ordered by mean grain zinc concentration. Zero and one indicate allele status at each position. Asterisks indicate alleles found exclusively in the top four Zn haplotypes. Bottom: boxplots showing grain Zn concentration associated with each genotype for SNPs unique to high Zn haplotypes.

Haplotypes for the four environment Zn QTL (*qZn7.2*) were further examined (Fig. 4). Ten haplotypes were identified in the panel (Fig. 4). The H40 haplotype was associated with high Zn (30.5 mg kg^−1^). Meanwhile, the most common haplotypes in *qZn7.2* was H1 associated with low Zn (21.2 mg kg^−1^).

#### QTL for agronomic traits

The QTL detected for and the four agronomic traits were distributed on all chromosomes: nine for DF, seven for GY, 28 for PH and twelve for TGW (Supplementary Table S4, Supplementary Fig. S1). The PVE by these QTL ranged from 2.9 to 14.7%. The QTL that explained the largest proportions of the PVE for each agronomic trait (with corresponding additive effects) were: *qDF2.2* (14.7%; 9.7 days), *qGY12.2* (7.2%, 150.3 kg ha^−1^), *qPH1.12* (12.8%; 49.3 mm), and *qTGW7.4* (7.7%; 4.3 mg).

Thirteen QTLs were consistently identified in two or more environments: one for DF (*qDF6.1*) eight for PH (*qPH1.1, qPH1.2*, *qPH1.4*, *qPH1.10, qPH1.11*, *qPH1.12*, *qPH1.13* and *qPH1.14*) and four for TGW (*qTGW4.1*, *qTGW6.1*, *qTGW7.3* and *qTGW12.1*). The QTL detected for GY, in contrast were only detected in single environments: two in IR13 and five in PR13.

#### Co-localisation of QTL

Co-localization among QTL for different traits were detected chromosomes 1, 2, 3, 6, 7, 8, 9, 10, 11 and 12 (Table 3). As expected, some highly correlated traits showed QTL that were co-located or in close proximity (within 100 kb). For examples, Zn had one common QTL with Cu on chr 1 and one with Mg on chr 3. Na and K shared a common QTL on chr 2, P and K shared one on chr 1.

**Table 3:** Summary of the co-localised QTL. *Env* environment (1 = IRRI 2012, 2 = IRRI 2013, 3 = PhilRice2012, 4 = PhilRice2013), *Chr* chromosome, *Start/End* physical Mb position of the linkage block, *Add. Eff* estimated additive effect, *PVE* phenotypic variation (%).

Chr	Co-located QTL	QTL	Env	Start	End	− log_10_P	PVE%	Add. Eff
1	*qK1.2, qP1.1, qPH1.1*	*qP1.1*	1	27.25	27.38	5.6	4.8	528.0
		*qK1.2*	1	27.29	27.33	5.7	4.3	443.5
		*qPH1.1*	1	27.25	27.29	6.1	3.0	28.8
		*qPH1.1*	4	27.25	27.29	7.8	3.9	19.2
1	*qCu1.1, qZn1.2, qPH1.10*	*qPH1.10*	1	38.48	38.56	6.2	8.1	50.6
		*qPH1.10*	2	38.22	38.56	8.7	12.8	49.3
		*qPH1.10*	4	38.39	38.63	7.3	10.6	41.8
		*qZn1.2*	1	38.28	38.43	6.0	9.1	2.9
		*qCu1.1*	1	38.36	38.46	5.3	10.2	1.2
2	*qK2.1, qNa2.2*	*qNa2.2*	4	19.69	19.71	5.5	8.0	5.2
		*qK2.1*	4	20.11	20.26	6.3	4.8	246.5
2	*qK2.2, qMn2.1, qDF2.2*	*qMn2.1*	2	33.94	33.97	6.8	5.7	8.2
		*qK2.2*	1	34.21	34.26	5.8	4.1	436.5
		*qDF2.2*	4	34.44	34.45	7.9	14.7	9.7
2	*qB2.1, qNa2.4*	*qNa2.4*	3	35.37	35.42	6.3	6.1	4.7
		*qB2.1*	3	35.67	35.77	6.1	10.9	0.6
3	*qB3.1, qCa3.1, qCo3.1, qMn3.1, qMo3.1, qTGW3.1*	*qMo3.1*	3	15.77	15.91	6.3	8.1	0.1
		*qCo3.1*	3	16.32	16.36	6.3	7.6	0.0
		*qMn3.1*	2	16.45	16.50	5.2	4.6	10.0
		*qB3.1*	2	16.46	16.50	6.7	7.8	5.9
		*qCa3.1*	1	16.66	16.92	6.0	9.5	15.1
		*qTGW3.1*	4	16.73	16.92	5.2	6.0	1.4
		*qCa3.1*	4	16.80	16.90	5.7	5.1	13.5
3	*qCo3.3, qFe3.2*	*qFe3.2*	1	34.98	35.01	5.7	5.2	3.4
		*qCo3.3*	2	35.15	35.21	5.5	4.5	0.0
6	*qDF6.3, qTGW6.1*	*qDF6.3*	2	22.53	22.54	5.9	5.2	16.9
		*qTGW6.1*	2	22.54	22.54	6.2	6.6	3.7
		*qTGW6.1*	4	22.54	22.54	5.6	5.7	3.3
7	*qCo7.1, qZn7.2*	*qCo7.1*	2	29.23	29.37	5.5	4.5	0.0
		*qCo7.1*	3	29.27	29.34	5.4	5.1	0.0
		*qCo7.1*	4	29.26	29.35	7.2	6.2	0.0
		*qZn7.2*	2	29.26	29.43	6.3	5.0	4.9
		*qZn7.2*	4	29.26	29.33	6.4	5.1	3.9
		*qZn7.2*	3	29.26	29.42	6.9	8.0	2.9
		*qZn7.2*	1	29.26	29.41	6.1	5.2	7.3
8	*qCo8.2, qMg8.1, qNa8.2, qPH8.2*	*qMg8.1*	3	19.62	19.77	6.4	7.4	146.3
		*qCo8.2*	1	20.04	20.14	6.5	7.2	0.0
		*qPH8.2*	4	20.23	20.31	6.8	5.2	30.0
		*qNa8.2*	3	20.67	20.72	8.5	6.9	4.5
9	*qNa9.1, qZn9.1*	*qNa9.1*	4	0.41	1.16	9.4	9.8	7.2
		*qZn9.1*	4	0.66	0.78	5.4	4.2	2.5
10	*qMn10.1, qMo10.2*	*qMo10.2*	3	5.17	5.28	5.4	4.4	0.1
		*qMo10.2*	2	5.17	5.36	7.1	7.5	0.6
		*qMn10.1*	2	5.17	5.22	5.4	3.8	7.0
10	*qMo10.4, qGY10.1*	*qGY10.1*	2	16.81	17.20	5.8	5.1	168.7
		*qMo10.4*	4	16.84	16.99	5.1	4.1	0.1
11	*qNa11.4, qMn11.1*	*qMn11.1*	3	26.88	26.95	6.0	4.8	4.1
		*qNa11.4*	4	26.96	27.19	6.2	6.0	4.9
12	*qB12.2, qZn12.1*	*qB12.2*	3	9.29	9.43	5.5	6.7	0.9
		*qZn12.1*	4	9.76	9.76	5.3	4.3	2.7
12	*qB12.3, qMo12.9*	*qMo12.9*	2	14.15	14.16	6.1	7.3	0.6
		*qB12.3*	3	14.21	14.39	5.7	7.8	0.9

All agronomic traits had co-located QTL with those of the grain elements (Table 3). DF shared a common QTL with K and Mn (chr 2). GY had co-located QTL with Mo (chr 10). TGW shared a common QTL with B, Ca, Co and Mo (chr 3). The QTL for PH were co-localized with those for Co, Cu, K, Mg, Na, P and Zn on chr 1 and 8.

The two genomic regions that harboured the highest number of QTL were on chrs 3 and 8 (Table 3). The region around 15.9–16.6 Mb on chr 3 contained QTL controlling five elements: B, Ca, Co, Mn and Mo (*qB3.1, qCa3.1, qCo3.1, qMn3.1, qMo3.2*) and TGW (*qTGW3.2*). On chr 8, the region between (19.6–20.6 Mb) had the QTL controlling the concentrations of Co, Mg, Na and PH (*qCo8.2, qMg8.1, qNa8.1, qPH8.1*).

#### Candidate genes

The physical positions of trait-associated markers were used to locate genes that were either linked to or in close proximity (within 300 kb) of the most significant SNPs. Supplementary Table S5 lists the potential candidate genes linked to the stable QTL (over three or more environments) and the major QTL clusters (on chrs 3 and 8). There were three main groups: (i) genes involved in metal transportation processes such as Zinc transporter (*ZIP2*), High-affinity potassium transporters (*HKT1, HKT9*), Nicotianamine synthase 3 (*NAS3*), Heavy-metal transport/detoxification, heavy-metal ATPase; (ii) genes controlling grain development such as Sucrose synthase (*SUS1*), Granule-bound starch synthase 1 (*GBSS1*), grain size (*GS3*) and (iii) genes controlling plant phenology such as Flowering-time locus (*FLT7*), Heading response regulator, senescence protein, No apical meristem protein (*NAC* factor).

## Discussion

### Phenotypic variation

The control of macro- and micro-nutrient homeostasis in plants have been extensively studied, however the loci that control natural ionomic variations in the grain are still largely undetermined in rice^31–33^. GWAS has been a powerful to dissect complex traits in plants^31^, however there are several factors that can limit the success of using GWAS to study the rice ionomes. These limiting factors include the relatively little variation in plant elemental concentrations and the often-substantial environmental effect^34,35^. In plant the transport and homeostasis of essential mineral nutrients are highly regulated processes as they require adequate levels of these essential nutrients for their growth and reproduction, while at the same time excess accumulation can also be detrimental to cell growth^36^.

The rice panel used in this study represents an excellent resource for genetic diversity covering a wide geographical and ecological variation in rice germplasm^25,26,30^. This diversity promising a large number of haplotypes is advantageous, however the effects of population structure need to be accounted for, in this case through a mixed model approach. The high density of SNPs (∼ 17 SNPs per kb on average) in our GWAS panel also facilitates high-resolution mapping with the loci were generally obtained within ∼ 100 kb, much higher than those obtained using linkage mapping approach^37^. This high resolution makes it possible to identify plausible candidate genes for a number of loci identified by GWAS using the information about the functional gene annotation or their orthologous genes in other plant species^38,39^.

The group of elements that showed relatively low levels of variation for the grain element concentrations consisted of four essential macronutrients (K, Mg, P, Ca) and four essential micronutrients (Cu, Fe, Mn and Zn) (Table 1, Fig. 2). These results indicate that the homeostasis of these elements is under relatively tight regulation. Previous research has shown that plants have evolved regulatory mechanisms to control the internal fluctuation of the essential nutrients to maintain their concentrations within narrow ranges for optimal growth, development and seed production^40,41^. Significantly larger variations were found for the concentration of the second group consisting of the three essential micronutrients B, Co and Mo and Na (Na is a functional but nonessential element^42^). It is likely that the elements in the second group were under less pressure to regulate their concentrations (unless they approach toxicity levels), thus having more relaxed control mechanisms. The differences in these control mechanisms exist not only among genotypes, they can also vary temporally and spatially within a given plant. Because this regulatory variability exists, it would appear that enhancing the micronutrient density of edible plant components through the manipulation of physiological processes is an achievable goal. The high heritability values of the nine grain elements also indicate that the contribution of genotypic variance to the total phenotypic variance was significant for these traits. Similar results were reported in previous studies^11,43,44^. Thus, direct selection for these elements may be a practical approach for trait improvement.

Our study shows significant variations in agronomic and grain element traits between four growing environments (Table 1). The most substantial differences were observed between the wet and the dry seasons and this might be because the wet season generally had lower temperature and higher rainfall during the period of vegetative and reproductive growth stages (Supplementary Table S6). These factors have been shown to influence flowering time in cereals^45^, which in turn would affect grain yield and grain nutrient levels^46,47^. The differences in soil chemistry, soil moisture and field management (including fertiliser application) (Supplementary Table S6) might also be the attributing factors to the variation between experimental sites.

### Trait correlation and QTL clusters

All twelve element concentrations in the grain were negatively correlated with grain yield in at least two environments using the Spearman’s rank correlation method (Supplementary Table S2). Six elements including Fe, K, Mg, Na, P and Zn had negative correlations with grain yield in all four environments and the highest correlation coefficients were found with Fe, Mg and P (r: − 0.39 to − 0.52). The negative correlations between grain yield and grain element concentrations are not uncommon in rice and have been reported in past studies for K, Mg, Mn, P, and Zn^11,20^. This likely reflects the dilution effects of increasing grain mass on the elemental concentrations. Minimizing the effect of grain yield for genetic mapping may be a required corrective measure in determining the genetics controlling this element accumulation in the grain, which would benefit breeding for rice lines with high nutrient concentrations. In our study, despite having strong negative correlations with all elements, grain yield had only one co-located QTL with Mo concentration on chr 10 (16.8 Mb) in one environment (PR13). This suggests that selection to enhance these grain elements at the identified loci is not likely to incur a yield penalty.

PH had consistent positive correlations with Co, Ca K and Zn; negative correlations with Cu and Mo and no correlations with Fe or Mn in three or more environments. As expected, PH QTL were located with those of Co, Ca, Na and Zn on chr 1 and 8. In theory, a taller plant will have more biomass and hence is able to accumulate higher levels of minerals during vegetative growth, which then becomes a larger source for remobilization of the stored minerals from leaves when they senesce at grain filling^48,49^.

Strong positive correlations between Co, Cu, Fe, K, Mg, Mn, and P were observed in three or more environments. This could be explained by an overlap in mechanisms to uptake and transport these elements within the plant. There have been several studies that reported correlations between different trace minerals^21,44,50^ and between essential minerals and toxic elements^21^. Genetic mapping has also been attempted to elucidate the genetic basis underlying these correlations in rice and other cereals^51–53^. Previous studies suggested that gene pleiotropy and QTL co-localization played a role in the correlations among trace minerals^21,44,51^. Similarly, several correlated traits were associated with the same QTLs either in the same or in a different environment in this study (Table 3). The results confirm that there is a highly complex genetic network controlling grain nutrition levels at multiple loci^19,54,55^. The co-localisations of Cu–Zn (chr 1), Co–Zn (chr 7), K–P (chr 1), K–Na (chr 2), K–Mn (chr 2) QTL support the possibility of simultaneous improvement of these elements in rice grain. Fe and Zn have been targeted for biofortification for decades^56,57^ and it is beneficial to explore the possibility to expand to other essential nutrients.

Despite of their strong correlations, P did not share any common QTL with either Zn, Fe or Mg. Thus, the selection for increasing the element concentrations of those at the loci is not likely to increase P concentration, which is desirable in relations to Zn and/or Fe bioavailability. In mature grain, P is mainly stored as phytate (myo-inositol-1,2,3,4,5,6-hexakisphosphate, InsP6), which has the ability to complex Zn and Fe forming insoluble complexes that cannot be digested or absorbed by humans^58^.

Two genomic regions contained the most QTL for element concentration on chrs 3 and 8 (Table 3). The region around 15.9–16.6 Mb on chr 3 harboured the QTL controlling five elements: B, Ca, Co, Mn and Mo (*qB3.1, qCa3.1, qCo3.1, qMn3.1, qMo3.2*) and TGW (*qTGW3.1*). Previous studies have also reported the association of this region with several grain element concentrations including Cd, Cu, Fe, Mn, P and Zn as well as grain length, thousand grain weight, grain yield and heading date^20,34,43,59^. There has not been any report of the QTL controlling B, Ca or Co concentration in this genomic region to date. On chr 8, the region between 19.6 and 20.6 Mb harboured the QTL controlling the concentrations of Co, Mg, Na and PH (*qCo8.2, qMg8.1, qNa8.1,* and *qPH8.1*). This region was also found to be associated with traits including Cd, Cu and Zn concentrations in the grain, Cu and Mg concentrations in the leaf, photosynthetic ability and plant height in previous studies^20,34,59,60^. This is the first time that a QTL for the grain Co and Na concentrations is being reported in this region. Overall, the two genomic regions on chr 3 and 8 that were associated with multiple elements could lead to the possibility for improvement of multiple nutrients simultaneously in rice breeding. However, grain yield and other developmental traits have also been mapped to the three regions in previous studies, suggesting that selection for higher grain nutrition may incur yield penalty and should be taken into consideration.

### Stable QTL

For QTLs to be highly effective within breeding programs, they must explain a significant proportion of the variation and be stable across environments and populations^31^. The stability of the QTL in our study was investigated over four environments. Among the QTL detected for grain elements, *qZn7.2* associated with Zn concentration on chr 7 was consistent in all four environments. The QTL with consensus in three environments were *qCo7.1* and *qK6.1*, associated with Co and K concentrations, respectively in three dry growing seasons. The two-environment QTL were found for eight traits including B, Ca, Co, Fe, K, Mo, Na and Zn concentrations. Interestingly, the four-environmental *qZn7.2* and the three-environmental *qCo7.1* were co-located on chr 7 (~ 29.26 Mb). The QTL accounted for approximately 5–8% and 4–6% of the variation in Zn and Co concentrations, respectively. The alleles associated with increased Zn and Co concentrations were present in less than 20% of the panel accessions indicating this was a rare allele, probably originating from an uncommon genetic pool. Not only was this QTL highly stable in our study, but it has also been identified in different genetic backgrounds. For example, significant QTL for grain Zn and Fe concentrations were reported in this genomic region on chr 7 (~ 29 Mb) in a Multi-parent Advanced Generation Intercross (MAGIC) population^43^ and a mapping population consisting of F_6_ recombinant inbred lines (RILs) derived from the cross Madhukar × Swarna^55^. Thus, our results reinforce the significance of the loci in controlling grain Zn density and affirm its potential as a strong target for Zn biofortification. Other traits that have been linked to this genomic region were grain inorganic P concentration^52^ and heading date^61^ which may have to be taken into account for breeding purposes.

The three-environment QTL *qK6.1* was located on the top of chr 6. This genomic region also harboured QTL for K, Cu and Zn concentrations and heading date in previous studies^11,20,62^. There has not been any QTL for grain yield reported in either of the genomic regions on chr 6 and 7 indicating that they are promising targets for improving Zn, Co and/or K concentration without yield penalty.

There was no stable QTL detected for grain yield or Cu, Mg, Mn and P concentrations. This is likely attributed to the large effect of the environmental conditions on the traits. For example, factors such as temperature, rainfall and/or soil chemistry could influence the bioavailability of these ions from the soil, which in turn affecting the mechanisms that plants would take for uptake, long-distance transport and remobilization^34,63^. Our results show significant environmental effects on the genetics controlling grain nutrient levels, which have also been reported in previous studies in wheat and rice^34,64^. Although consensus QTL can generally be considered as more favourable for marker-assisted selection, some QTL detected in one environment may lead to important discoveries.

### Key candidate genes underlying the QTL clusters on chromosomes 3 and 8

Underlying the QTL clusters, there were key genes that control plant phenology, grain development and metal transportation (Table 4 and Supplementary Table S5). Three genes involved in the processes of starch synthesis and grain filling were linked to the QTL cluster on chr 3. These genes were *SUS1* (*Os03g0401300*), *GS3* (*Os03g0407400*) and GS5 (*Os03g0393300*). *SUS1* (linked to *qCo3.1*) encodes a sucrose synthase (Sucrose-UDP glucosyltransferase) responsible for the biosynthesis of starch within the endosperm. Overexpression of this gene in transgenic rice lines led to increased GY (per plant) and TGW^65^. *GS3* (linked to *qCa3.2* and *qTGW3.1*) and *GS5* (linked to *qMo3.2*) encode a transmembrane protein and a putative serine carboxypeptidase, respectively^66,67^. Natural variations in either of these genes were found to play important roles in regulating grain filling and final grain size and weight^67–69^. The results indicate a link between the processes of starch synthesis/grain filling and grain element accumulation. The transfer route of micronutrients (such as Fe and Zn) into the grain is thought to be similar to that of sucrose^70,71^. In transgenic wheat lines overexpressing a sucrose transporter gene, there was an increase in grain yield as well as a 20–40% increase in grain Fe and Zn concentrations^78^. The functionality of those genes in relation to controlling grain nutrient elements such as Ca, Co and Mo in rice, will require further studies to elucidate.

**Table 4:** Genes included in the localized region delimited by the most significantly associated SNPs with element concentrations. Genes involved in metal transporting are coded in bold, flowering in italics, starch synthesis/grain size in bolditalics. Gene annotation information is from https://rapdb.dna.affrc.go.jp/download/irgsp1.html.

GeneID	Chr	Loc (Mb)	Description	Linked QTL
Os03g0392600	3	15.8	*OsSCP14*—Serine carboxypeptidase homologue	*qMo3.2*
Os03g0395000	3	15.9	Stroma-localized heme oxygenase 2	*qCa3.1*
Os03g0401366	3	16.3	***OsSUS1; Sucrose synthase (EC 2.4.1.13)***	*qCo3.1*
Os03g0407400	3	16.7	***GS3, Regulator of grain size and organ size***	*qCa3.2, qTGW3.1*
Os03g0410100	3	16.9	SUMO protease protein	
Os03g0411800	3	17.0	**Zinc transporter 2 (*OsZIP2*)**	
Os03g0412300	3	17.0	**Heavy metal transport/detoxification protein**	
Os03g0412800	3	17.1	Glucose-6-phosphate dehydrogenase precursor	
Os03g0413400	3	17.1	Glycosyl transferase, family 8 protein	
Os06g0129400	6	1.6	**Vacuolar phosphate efflux transporter, *OsSPX-MFS3***	*qB6.1*
Os06g0130400	6	1.6	***ACC synthase; starch in endosperm***	*qK6.1*
Os06g0131500	6	1.7	Glucan endo-1, 3-beta-glucosidase 7	
Os06g0131700	6	1.7	No apical meristem (NAM) protein	
Os06g0133000	6	1.8	***Granule-bound starch synthase 1 (OsGBSS1)***	
Os06g0701600	6	29.5	**High-affinity K + transporter 9; *OsHKT9***	*qZn6.1*
Os06g0701700	6	29.5	**Na+/K+; high-affinity K + transporter 1 *OsHKT1***	
Os07g0688000	7	29.2	***Metallophosphoesterase***	*qCo7.1, qZn7.2*
Os07g0689600	7	29.3	***OsNAS3* Nicotianamine synthase 3**	
Os07g0690300	7	29.4	Zinc finger, RING/FYVE/PHD-type	*qZn7.2*
Os07g0690800	7	29.4	Phytochelatin synthase 12	
Os07g0690900	7	29.4	Glycosyl-phosphatidyl inositol-anchored	
Os07g0691100	7	29.4	Pectin methylesterase 6	
Os07g0692900	7	29.5	Ubiquitin-activating enzyme E1	*qZn7.2, qCu7.1*
Os07g0693100	7	29.5	Pyruvate decarboxylase isozyme 3 (EC 4.1.1.1);	
Os07g0694000	7	29.5	Phosphoinositide phospholipase C, Salt tolerance	
Os07g0694700	7	29.6	Ascorbate peroxidase, Carbohydrate metabolism	
Os07g0695100	7	29.6	*Heading response regulator; Long-day repression*	
Os08g0410500	8	19.6	**Carbohydrate transporter/sugar porter/transporter**	*qMg8.1, qPH8.1*
Os08g0414700	8	19.8	Trehalose-6-phosphate synthase	*qPH8.1*
Os08g0421700	8	20.2	Zinc finger, CCHC-type	*qCo8.2*
Os08g0423600	8	20.3	Carbonic anhydrase	*qPH8.3*
Os08g0425300	8	20.4	Endoglucanase 21	

On chr 8, the gene *Os08g0430500* codes for a 14-3-3 protein (Florigen receptor) involved in controlling flowering time in rice^72^. Flowering, grain filling and whole-plant senescence are processes that are highly important in determining grain weight, yield and quality parameters such as grain protein content (GPC) and grain micronutrient including Fe, Mn and Zn levels in cereals^46,47^.

Metal transporter genes were linked to the QTL clusters on both chrs 3 and 8. On chr 3, two genes, *Os03g0411800* (*OsZIP2*) and *Os03g0412300* (a heavy metal transport/detoxification) were located within the QTL for Ca concentration and TGW. On chr 8, the gene *Os08g0422200* (linked to *qPH8.3* and *qCo8.2*) codes for a Cation efflux protein, namely MTP12 (Metal tolerance protein)*.* All of the three transporters have broad substrate transport activity (transporting Zn^2+^, Cd^2+^ Co^2+^, Cu^2+^, Fe^2+^, Mn^2+^, Ni^2+^)^73–75^, which may explain their involvement with the transport of multiple elements under the QTL clusters. The ZIP transporter family mostly mediates metal ions influx to the cytoplasm of root cells and some members (*OsZIP1, 2, 4, 5, 7, and 8*) were highly induced by Zn deficiency^73,76^. The Cation efflux protein family is involved mainly in the compartmentation of metal ions into organelles such as vacuoles at high concentrations^77^. Mineral uptake and transportation in rice has been revealed being a complex process that involved the combined actions of several transporter genes^78,79^. The genes being identified in this study would be potential candidates for further studies to improve essential nutrients in the rice grain.

### Key candidate genes underlying the stable QTL on chromosomes 6 and 7

Located within the markers flanking the four-environment QTL for Zn (*qZn7.2*), there was a prominent potential candidate gene *OsNAS3* (*Os07g0689600*) coding for nicotianamine synthase 3 (Table 4). Nicotianamine synthase (NAS) is the enzyme responsible for production of nicotianamine (NA), a metal chelator for the internal transport of diverse metals, including Cu, Fe, Mn and Zn^80^. In rice, NA bound to Zn in phloem is supposed to avoid Zn immobilization in the alkaline conditions of the phloem sap, thus playing a vital role in intercellular and long-distance transport of Zn to maintain Zn homeostasis in plants^48,81^. Rice possesses three *NAS* genes, namely *OsNAS1-3*^82^. Overexpression of each *NAS* gene led to significant increases of Fe and Zn levels in the rice grain^83,84^ implying that they can all be targets for improving Zn and Fe concentrations in rice grain. The fact that *NAS3* gene was linked to the stable Zn and Co QTL implies the important roles of the phloem transport processes for Zn and possibly also for Co from vegetative tissues into the grain. The presence of SNPs specific to high Zn haplotypes within the *OsNAS3* promoter^43^ suggests that the effect of q*Zn-7.2* may be achieved through modulating expression of this gene.

On chr 6, there was one gene coding for granule-bound starch synthase *GBSS1* (*Os06g0133000*) located within the three-environment QTL *qK6.1*. This enzyme is involved in starch synthesis during grain filling, specifically being responsible for the synthesis of amylose and building the final structure of amylopectin^85^. Similar to the cases of *SUS1* (on chr 3), the results here propose an important relationship between the processes of starch synthesis/grain filling and nutrient upload into the grain.

In conclusion, the rice diversity panel used in this study proved to be a useful resource for association mapping of rice grain nutrition with significant variation observed and QTL detected for all traits. Co-localizations of QTL for multiple grain element concentrations was found, and particularly those on chrs 3 and 8 open the opportunity for enhancing multi elements simultaneously. Consistent QTL across environments were identified, particularly the four-environment QTL for Zn (*qZn7.2*). This QTL had been reported previously, indicating its stability in different genetic backgrounds, and is a strong candidate for being used in breeding for higher Zn concentration. Multiple candidate genes were identified, which can potentially play various roles in controlling mineral accumulations in rice grain including *NAS3*, *SUS1 and GBSS1*. Further gene functionality studies would be helpful to validate the significance of the candidate genes in breeding for higher micronutrient content in rice grains.

## Materials and methods

### Plant material and field trial

Field trials were planted at two sites in the Philippines: (i) IRRI (14° 15′ N 121° 27′ E) during the 2012 wet season and 2013 dry season and (ii) PhilRice (15° 67′ N 120° 89′ E) during the 2012 and 2013 dry seasons. The wet season was sown in June and the dry season was in January each year. Each field trial comprised three replicates planted in a randomized complete block design. The accessions were sown at the same time and grouped by previously estimated heading date and plant height to facilitate measurements. Detailed description of the experimental design, watering and fertiliser regime, disease and weed management are included in Supplementary Table S6.

### Phenotyping

At maturity, plants of from the middle two rows (excluding the border rows) were harvested to assess yield (14% moisture) and thousand grain weight (TGW) following standard procedure^85^. Days to flowering (DF) was assessed as the interval between the date of sowing and the date when panicles of 50% of plants per plot were fully exerted. Plant height (PH) was measured from the base of the root–shoot junction to the tip of the flag leaf, which was manually straightened to be aligned with the culm.

### Grain nutrient analysis

Representative samples of about 250 g of mature grains collected from each plot were analysed for grain nutrient concentration at the Flinders Analytical Laboratory (Flinders University, Australia). The twelve grain elements being analysed included boron (B), calcium (Ca), cobalt (Co), copper (Cu), iron (Fe), potassium (K), magnesium (Mg), manganese (Mn), molybdenum (Mo), sodium (Na), phosphorus (P) and zinc (Zn). Approximately 0.3 g of each grain sample (oven dried at 80 °C for 4 h) was digested with a closed tube acid digestion as described^86^. Grain element concentrations were determined using inductively coupled plasma mass spectrometry (ICP-MS 7500x; Agilent, Santa Clara, CA) following the method described in Ref.^87^. A blank and a certified reference material (CRM; NIST 1567a wheat flour) were included in each digestion batch for quality assurance. The element concentrations were expressed on a dry weight basis. Contamination with soil was monitored by analysis for aluminium and titanium.

### Statistical analysis

Statistical analyses were conducted by using R statistical software (ver. 3.6.0) and Genstat18^88^. The non-linear correlation between all traits was determined within each seasonal dataset using the Spearman rank correlation method as implemented in the *corr.test* function from the *psych* package^89^. The significance of the correlations was determined with two-sided test of the correlations against 0 at a probability of 0.05. The means of different seasons were compared using one-way ANOVA at a 0.05 level of probability. The frequency distributions of grain mineral concentrations and TGW were demonstrated using Histogram.

### Genotypic data, population structure and linkage disequilibrium

Genotypic data describing 5.2 million biallelic SNPs in a rice reference panel covers 233 genotypes from the PRAY Indica panel^30^ and was used in this current study. The number of independent markers in the genotypic data was estimated according to the autocorrelation method described by Sobota et al*.*^89^. Briefly, genotypic data was split by chromosome and recoded to represent the number of minor alleles at each locus for each individual. An autoregressive model was fit to each individual to estimate the number of independent markers. This number was averaged for each chromosome and the final number of independent markers derived by summing all chromosomes.

Principal component analysis was conducted using PLINK 1.9^90^ to identify population substructure and identify non-Indica individuals.

A kinship matrix was constructed using the IBS model in *emmax*^91^ to describe cryptic relatedness in the population.

### GWAS

Normality of phenotypic distribution was assessed using the Shapiro–Wilk test implemented in R (R Core Team 2018) using a significance threshold of p < 0.05. Where possible, phenotypes found not to be normally distributed were transformed to normality using the following transformations: square root, cube root, natural log, inverse cube root, inverse square root, inverse. GWAS were performed for all transformations up to and including the first to be normally distributed.

GWAS were performed utilising a mixed linear model (MLM) in *emmax*, incorporating kinship plus up to two principal components to account for population structure.

In the mixed model, principal components and family kinship were included

$${\text{Y}} = {\text{X}}_{\alpha } + {\text{ Q}}_{\beta } + {\text{K}}_{\mu } + {\text{ e,}}$$

where Y represents the vector of phenotype, X represents the vector of SNPs, Q is the PCA matrix and K is the relative kinship matrix. X_α_ and Q_β_ are the fixed effects, and K_μ_ and e represent random effects. The Q and K matrices help to reduce the spurious false positive associations. Correction for population structure (Q) substantially reduces the false positives but it sometimes eliminates true positive associations due to overcorrection. Therefore, the optimal number of principal components was estimated for each trait before incorporating them for MLM tests, based on the forward model selection method using the Bayesian information criterion. This method helps to control both false-positive and -negative associations more effectively although it cannot eliminate both completely.

The lambda genomic inflation factor was determined for each association analysis using the regression method of the *estlambda* function from the *GenABEL* R package^92^. In comparing multiple association models applied to a single trait, an inflation factor closest to one signified the best analysis.

A significance threshold α = 0.05 was used for association mapping, but was adjusted using the Bonferroni approach considering the estimated number of independent markers:

$$\alpha_{adj} = \, \alpha /n,$$

where α is the unadjusted significance threshold and n is the number of independent markers in the population.

Quantitative trait loci (QTLs), regions containing SNPs associated with phenotypes, were defined as described by McCouch et al*.*^29^. A QTL was defined as any region containing one SNP with − log_10_(p) > − log_10_ (α_adj_) flanked by markers with − log_10_ (p) > 4 on each side and within 100 kb.

### Candidate gene and haplotype analysis

The physical locations of SNPs were identified based on the Rice Annotation version of 7.0 of variety Nipponbare from Michigan State University. Considering that the LD decay distance in XI accessions is about 100 kb^93^, significant SNPs located to a region of less than 100 kb were treated as one locus. The annotations of genes located within LD blocks were obtained from the Os-Nipponbare-Reference-IRGSP-1.0 rice genome database (https://rapdb.dna.affrc.go.jp/download/irgsp1.html).

Haplotype analysis of candidate genes plus 1.5 kb upstream was performed in R using the imputed SNP dataset.

### Trait heritability

Trait heritability and genotype × environment interactions were investigated using additive main effects and multiplicative interactions (AMMI) model in GenStat^88^. The computed variance components were used to estimate broad-sense heritability across the four environments tested using the formula described by Velu et al*.*^94^:

$$H^{2} = \, \sigma g^{2} /\left( {\sigma g^{2} + \, \sigma ge^{2} + \, \sigma e^{2} / \, rl} \right),$$

where *H*^2^ is the broad-sense heritability*, σg*^2^ is the genotypic variance, *σge*^2^ is the genotype × environment variance, and *σe*^2^ is the residual error variance for *r* replicates and *l* locations.

### Ethics declarations

The authors declare that the experimental research and field studies on plants in this study including the collection of plant material comply with relevant institutional, national, and international guidelines and legislation.

## Supplementary Information

Supplementary Information.

## Abbreviations

AM: Association mapping
B: Boron
Ca: Calcium
Chr: Chromosome
Co: Cobalt
Cu: Copper
CV: Coefficient of variation
DF: Days to 50% flowering
Fe: Iron
GWAS: Genome-wide association study
GY: Grain yield
ICP-MS: Inductively coupled plasma mass spectrometry
IRRI: International Rice Research Institute
K: Potassium
LD: Linkage disequilibrium
Mg: Magnesium
MLM: Mixed-linear model
Mn: Manganese
Mo: Molybdenum
MTA: Marker-trait association
Na: Sodium
P: Phosphorus
PCA: Principal component analysis
PH: Plant height
PRAY: Phenomics of rice adaptation and yield
SNP: Single nucleotide polymorphism
TGW: Thousand grain weight
Zn: Zinc
