# Supplementary material for: A high-resolution genome-wide association study of the grain ionome and agronomic traits in rice Oryza sativa subsp. indica
Source: Sci Rep. 2021 Sep 28;11:19230. doi: 10.1038/s41598-021-98573-w (PMC8478900; doi:10.1038/s41598-021-98573-w)

Supplementary data

A high-resolution genome-wide association study of the grain ionome and agronomic traits in rice *Oryza sativa* subsp. *indica*

Suong T. Cu[1]*, Nicholas Warnock[2]*, Julie Pasuquin[3], Michael Dingkuhn[4] and James Stangoulis[1]

Table S1: Correlations between four environments (IR12: IRRI 2012, IR13: IRRI 2013, PR12: PhilRice 2012, PR13: PhilRice 2013) for each trait and their significant levels (*** P<0.001, **P<0.01 and *P <0.05. Correlation coefficient values were determined by Spearman's correlation analysis. DF: days to flowering, PH, plant height; GY, grain yield

	**IR12&IR3**	**IR2&PR12**	**IR12&PR13**	**IR3&PR12**	**IR13&PR13**	**PR12&PR13**
**Ca**	0.68***	0.58***	0.71***	0.71***	**0.83*****	**0.80*****
**Mo**	0.76***	0.63***	0.60***	0.62***	0.59***	0.67***
**Mn**	0.64***	0.50***	0.63***	0.60***	0.6***5	**0.69*****
**K**	**0.67*****	0.51***	**0.65*****	0.50***	0.65***	0.56***
**PH**	0.83***	0.51***	0.80***	0.56***	**0.93*****	0.55***
**Zn**	0.60***	0.44***	0.55***	0.59***	**0.75*****	0.66***
**Fe**	0.52***	0.27***	0.45***	0.52***	**0.71*****	0.57***
**Co**	0.59***	0.30***	0.40***	0.64***	0.62***	**0.71*****
**P**	0.62***	0.30***	0.51***	0.53***	**0.71*****	0.57***
**Mg**	**0.58*****	0.20**	0.42***	0.43***	**0.59*****	0.37***
**Na**	0.35***	**0.50*****	0.41***	0.25**	0.43***	**0.53*****
**Cu**	0.25**	0.12	0.02	0.56***	0.47***	**0.60*****
**DF**	0.32***	0.07	0.13	**0.85*****	**0.86*****	**0.87*****
**GY**	0.36***	-0.01	0.10	-0.08	0.14	**0.41*****
**B**	0.38***	0.31***	-0.01	0.19*	0.14	0.08

Table S2: Correlations between all traits within each environment (A: IRRI 2012, B: PhilRice 2012, C: IRRI 2013, D: PhilRice 2013). Correlation coefficient values were determined by Spearman's correlation analysis. All the correlations coloured in dark, medium and light orange/blue are significant at P<0.001, P<0.01 and P <0.05, respectively. DF: days to flowering, PH, plant height; GY, grain yield, TGW: thousand grain weight

A: IR12

	B	Ca	Co	Cu	Fe	K	Mg	Mn	Mo	Na	P	Zn	DF	GY
**Ca**	0.33	-												
**Co**	0.00	0.13	-											
**Cu**	-0.07	0.11	-0.02	-										
**Fe**	0.08	0.27	**0.45**	0.20	-									
**K**	0.29	0.20	0.37	0.07	0.24	-								
**Mg**	0.24	0.32	0.21	0.23	**0.42**	**0.48**	-							
**Mn**	0.09	**0.41**	0.08	0.16	0.27	0.13	0.16	-						
**Mo**	-0.04	-0.01	0.08	0.01	0.27	0.09	0.14	0.21	-					
**Na**	0.15	0.12	0.07	0.11	-0.01	0.39	-0.05	0.07	0.01	-				
**P**	0.27	0.30	0.36	0.21	**0.52**	**0.69**	**0.85**	0.23	0.24	0.08	-			
**Zn**	0.25	0.10	0.06	**0.52**	0.26	0.37	0.36	0.17	0.03	0.25	**0.45**	-		
**DF**	0.13	-0.08	**-0.49**	0.19	**-0.42**	0.02	-0.14	-0.08	-0.22	0.30	-0.19	**0.47**	-	
**GY**	-0.20	-0.20	-0.21	-0.04	**-0.40**	**-0.52**	-0.38	-0.16	-0.21	-0.25	**-0.51**	-0.29	0.03	-
**PH**	0.38	0.24	0.02	0.03	-0.28	0.25	-0.02	-0.13	-0.32	**0.41**	0.05	**0.42**	**0.58**	-0.01

B: PR12

	B	Ca	Co	Cu	Fe	K	Mg	Mn	Mo	Na	P	Zn	DF	GY
**Ca**	0.23	-												
**Co**	0.09	0.26	-											
**Cu**	0.09	-0.12	-0.07	-										
**Fe**	0.04	0.31	0.29	0.38	-									
**K**	0.17	0.31	0.24	0.05	**0.41**	-								
**Mg**	0.10	0.32	0.26	0.24	**0.54**	**0.62**	-							
**Mn**	0.01	**0.41**	0.23	0.28	**0.47**	0.37	**0.40**	-						
**Mo**	-0.05	0.12	0.07	0.25	0.32	0.11	0.17	0.32	-					
**Na**	0.13	0.3	0.19	-0.22	0.29	0.35	0.17	0.24	0.01	-				
**P**	0.17	0.36	0.34	0.27	**0.59**	**0.74**	**0.89**	**0.45**	0.27	0.29	-			
**Zn**	0.19	0.18	0.37	0.3	**0.44**	0.34	0.39	0.31	0.25	0.20	**0.53**	-		
**DF**	-0.23	-0.37	-0.09	-0.15	**-0.40**	-0.12	-0.24	-0.21	-0.07	**-0.45**	-0.30	-0.10	-	
**GY**	-0.11	-0.07	0.02	**-0.48**	-0.37	-0.19	-0.35	-0.23	-0.30	-0.08	**-0.44**	-0.37	0.24	-
**PH**	0.1	0.24	0.33	**-0.40**	0.04	0.32	0.18	-0.03	-0.08	0.38	0.18	0.14	-0.03	0.18

Table S2

C: IR13

	B	Ca	Co	Cu	Fe	K	Mg	Mn	Mo	Na	P	Zn	DF	GY	PH
**Ca**	**0.54**	-													
**Co**	0.07	0.22	-												
**Cu**	-0.01	-0.24	-0.07	-											
**Fe**	0.06	0.23	0.32	0.03	-										
**K**	0.04	0.17	0.32	-0.17	0.27	-									
**Mg**	0.05	0.13	0.27	0.11	0.28	0.33	-								
**Mn**	0.36	**0.48**	0.22	0.04	0.25	0.04	-0.02	-							
**Mo**	0.05	0.12	0.14	0.14	0.33	0.18	0.05	0.37	-						
**Na**	-0.05	0.08	0.08	-0.21	0.16	0.32	-0.14	0.00	0.19	-					
**P**	0.13	0.26	0.40	0.01	**0.41**	**0.6**	**0.81**	0.09	0.24	0.04	-				
**Zn**	0.11	0.17	0.29	0.11	**0.54**	0.24	**0.45**	0.13	0.21	-0.14	**0.57**	-			
**DF**	-0.32	**-0.40**	-0.30	0.05	-0.27	0.00	-0.14	**-0.47**	-0.28	-0.01	-0.26	-0.18	-		
**GY**	0.02	-0.16	-0.30	0.16	-0.26	**-0.44**	-0.29	0.00	-0.08	-0.16	-0.40	-0.32	-0.04	-	
**PH**	-0.03	0.21	0.18	-0.39	0.11	0.30	0.21	-0.11	-0.20	0.02	0.33	0.34	0.09	-0.29	-
**TGW**	-0.18	-0.36	-0.06	0.07	-0.02	-0.02	-0.24	-0.03	0.06	-0.09	-0.18	0.04	-0.04	0.20	-0.01

D: PR13

	B	Ca	Co	Cu	Fe	K	Mg	Mn	Mo	Na	P	Zn	DF	GY	PH
**Ca**	-0.02	-													
**Co**	-0.24	0.25	-												
**Cu**	0.11	-0.10	0.01	-											
**Fe**	0.06	0.25	0.32	0.36	-										
**K**	-0.01	0.28	0.23	0.06	0.21	-									
**Mg**	-0.01	0.09	0.06	**0.40**	0.31	0.38	-								
**Mn**	0.05	0.36	0.19	0.28	0.34	0.21	0.17	-							
**Mo**	-0.04	-0.10	-0.05	0.09	0.09	0.04	0.07	0.02	-						
**Na**	-0.05	0.21	0.25	0.09	0.38	**0.49**	0.23	0.29	0.07	-					
**P**	-0.02	0.25	0.27	**0.43**	**0.44**	**0.60**	**0.73**	0.28	0.09	**0.45**	-				
**Zn**	-0.07	0.22	**0.41**	**0.40**	**0.41**	0.25	0.26	0.32	0.07	0.23	**0.50**	-			
**DF**	-0.25	-0.31	-0.13	-0.29	**-0.42**	-0.17	-0.25	-0.13	0.06	-0.36	**-0.44**	-0.02	-		
**GY**	-0.22	0.00	0.00	**-0.42**	**-0.42**	-0.15	-0.32	-0.19	-0.27	-0.33	-0.39	-0.17	0.39	-	
**PH**	-0.28	0.33	0.33	-0.24	0.02	0.25	-0.04	0.06	-0.16	0.09	0.13	0.31	0.13	0.38	-
**TGW**	0.04	-0.30	-0.02	0.09	0.02	-0.05	-0.14	-0.13	0.05	-0.03	-0.15	0.04	-0.09	0.03	0.03

Table S3: Summary of the QTL for the concentration of twelve elements (mg kg^{-1}) in mature grain of the PRAY panel grown in four environments (IR12: IRRI 2012, IR13: IRRI 2013, PR12: PhilRice 2012, PR13: PhilRice 2013) detected by mixed linear models. QTL detected in more than one environment are shaded. Chr, chromosome; Start/End: physical position of the linkage block; Add. Effect: estimated additive effect, PVE: phenotypic variation explained by the QTL (%). High All. Freq: Frequency of the higher value allele.

Trait	QTL	Env	No. env	Chr	Start Mb	End Mb	-log$_{10}$(P)	PVE (%)	Add. Effect	High All. Freq.
**B**	*qB1.1*	IR13	1	1	3.97	4.03	6.3	6.9	4.77	0.09
	qB2.1	PR12	1	2	35.67	35.77	6.1	10.9	0.56	0.91
	qB3.1	IR13	1	3	16.46	16.50	6.7	7.8	5.88	0.05
	qB4.1	PR13	1	4	0.06	0.41	7.0	7.2	2.54	0.09
	qB4.2	PR13	1	4	1.91	2.05	6.9	6.4	2.57	0.07
	qB4.3	IR12	1	4	29.88	30.03	5.6	11.8	0.96	0.89
	qB7.1	IR12	2	7	6.06	6.49	8.8	9.7	2.44	0.12
	qB7.1	IR13	2	7	6.06	6.49	8.9	11.5	4.03	0.16
	qB7.3	PR13	1	7	26.45	26.51	5.1	5.7	3.07	0.06
	qB7.4	PR13	1	7	27.59	27.74	5.4	4.6	2.64	0.06
	qB12.1	IR12	1	12	8.85	8.85	5.2	5.5	2.68	0.05
	qB12.2	PR12	1	12	9.29	9.43	5.5	6.7	0.91	0.93
	qB12.3	PR12	1	12	14.21	14.39	5.7	7.8	0.90	0.92
	qB12.4	PR12	1	12	16.40	16.66	5.9	7.2	0.93	0.93
	qB12.5	PR12	1	12	19.18	19.42	6.0	9.2	0.75	0.80
**Ca**	*qCa3.1*	IR12	2	3	16.66	16.92	6.0	9.5	15.12	0.56
	qCa3.1	PR13	2	3	16.80	16.90	5.7	5.1	13.52	0.41
	qCa4.1	IR13	1	4	19.12	19.19	5.5	6.1	9.24	0.30
	qCa8.1	IR12	1	8	5.27	5.34	5.4	7.0	12.57	0.18
	qCa11.1	IR13	1	11	16.28	16.34	5.4	4.0	16.42	0.93
	qCa12.2	PR12	2	11	27.83	27.83	5.2	4.7	6.03	0.66
	qCa12.2	PR13	2	11	27.83	27.83	5.2	3.6	7.89	0.68
**Co**	*qCo1.1*	PR13	1	1	13.78	13.78	5.6	5.3	0.01	0.81
	qCo3.1	PR12	1	3	16.32	16.36	6.3	7.6	0.01	0.25
	qCo3.2	IR13	1	3	31.13	31.22	5.2	5.6	0.01	0.64
	qCo3.3	IR13	1	3	35.15	35.21	5.5	4.5	0.02	0.05
	qCo4.1	IR13	1	4	0.58	0.71	5.3	5.2	0.01	0.09
	qCo6.1	IR12	2	6	4.23	4.38	5.6	5.7	0.02	0.18
	qCo6.1	IR13	2	6	5.59	5.66	5.1	4.6	0.01	0.59
	qCo6.2	IR13	1	6	9.90	9.94	5.2	4.9	0.02	0.18
	qCo7.1	IR13	3	7	29.23	29.37	5.5	4.5	0.02	0.14
	qCo7.1	PR13	3	7	29.26	29.35	7.2	6.2	0.03	0.14
	qCo7.1	PR12	3	7	29.27	29.34	5.4	5.1	0.02	0.14
	qCo8.1	PR13	2	8	3.55	3.56	5.5	12.4	0.00	0.09
	qCo8.1	PR12	2	8	3.55	3.57	6.2	15.7	0.00	0.90
	qCo8.2	IR12	1	8	20.04	20.14	6.5	7.2	0.02	0.09
	qCo12.1	IR12	1	12	1.01	1.03	5.4	6.1	0.01	0.30

Trait	QTL	Env	No. env	Chr	Start Mb	End Mb	$-\log_{10}(P)$	PVE (%)	Add. Effect	High All. Freq.
**Cu**	*qCu1.1*	IR12	1	1	38.36	38.46	5.3	10.2	1.17	0.90
	qCu4.1	IR13	1	4	27.32	28.04	8.5	10.4	1.43	0.82
	qCu12.1	PR13	1	12	23.40	23.45	5.3	5.6	0.58	0.89
**Fe**	*qFe1.1*	PR13	1	1	2.37	2.42	5.6	6.4	0.36	0.71
	qFe1.2	PR13	2	1	2.53	2.64	5.9	6.7	0.41	0.60
	qFe1.2	IR13	2	1	2.56	2.66	5.6	7.0	0.44	0.82
	qFe2.1	PR12	1	2	6.07	6.20	5.8	6.1	1.19	0.80
	qFe3.1	IR12	1	3	34.69	34.82	5.3	5.6	1.31	0.25
	qFe3.2	IR12	1	3	34.98	35.01	5.7	5.2	3.42	0.05
	qFe3.3	IR12	1	3	35.82	35.96	5.5	4.6	2.64	0.06
	qFe7.1	IR13	1	7	10.49	10.57	5.1	8.2	1.83	0.07
	qFe7.2	IR13	1	7	11.04	11.58	5.4	10.1	1.75	0.12
	qFe7.3	IR13	1	7	11.65	11.97	5.6	7.3	1.50	0.08
	qFe7.4	IR13	1	7	12.28	12.60	5.3	7.1	1.64	0.08
	qFe7.5	IR13	1	7	12.62	12.93	5.4	6.6	1.44	0.08
	qFe7.6	IR13	1	7	13.14	13.60	5.8	7.9	1.90	0.06
	qFe8.1	IR13	1	8	0.63	0.65	5.3	5.4	0.92	0.23
	qFe12.1	PR12	1	12	7.58	7.59	5.2	5.9	1.87	0.95
**K**	*qK1.1*	IR13	1	1	26.40	26.42	5.4	4.6	549.58	0.06
	qK1.2	IR12	1	1	27.29	27.33	5.7	4.3	443.45	0.06
	qK2.1	PR13	1	2	20.11	20.26	6.3	4.8	246.45	0.06
	qK2.2	IR12	1	2	34.21	34.26	5.8	4.1	436.48	0.06
	qK4.1	IR12	1	4	5.15	5.30	5.1	3.7	445.53	0.05
	qK4.2	PR13	1	4	12.54	12.59	5.8	7.4	184.56	0.37
	qK5.1	IR13	1	5	9.29	9.38	5.2	4.8	419.63	0.11
	qK6.1	PR13	3	6	1.59	1.83	7.8	6.2	255.44	0.13
	qK6.1	PR12	3	6	1.70	1.81	5.5	5.0	261.83	0.13
	qK6.1	IR13	3	6	1.70	1.83	6.0	4.5	233.69	0.13
	qK10.1	IR13	1	10	21.43	21.48	5.4	5.1	55.13	0.66
	qK11.1	IR13	1	11	18.73	18.75	5.2	4.9	222.91	0.30
**Mg**	*qMg3.1*	PR13	1	3	8.70	8.82	6.1	10.3	51.60	0.37
	qMg3.2	PR13	1	3	12.85	12.91	5.3	6.4	110.89	0.10
	qMg8.1	PR12	1	8	19.62	19.77	6.4	7.4	146.33	0.15
**Mn**	*qMn1.1*	IR13	1	1	29.33	29.34	5.4	7.3	9.11	0.94
	qMn1.2	PR12	1	1	30.74	30.89	5.2	5.6	3.70	0.15
	qMn2.1	IR13	1	2	33.94	33.97	6.8	5.7	8.17	0.10
	qMn3.1	IR13	1	3	16.45	16.50	5.2	4.6	9.98	0.06
	qMn6.1	PR12	1	6	0.12	0.26	5.7	6.3	2.01	0.78
	qMn8.1	IR13	1	8	8.81	8.91	6.3	6.0	8.86	0.11
	qMn9.1	IR12	1	9	10.15	10.38	7.4	10.5	2.95	0.51
	qMn9.2	IR12	1	9	10.48	10.54	5.6	8.6	2.34	0.55
	qMn10.1	IR13	1	10	5.17	5.22	5.4	3.8	7.01	0.09
	qMn11.1	PR12	1	11	26.88	26.95	6.0	4.8	4.08	0.09

Trait	QTL	Env	No. env	Chr	Start Mb	End Mb	-log$_{10}$(P)	PVE (%)	Add. Effect	High All. Freq.
**Mo**	*qMo1.1*	IR13	1	1	28.52	28.84	6.6	7.5	0.33	0.22
	qMo3.1	PR12	1	3	15.77	15.91	6.3	8.1	0.11	0.09
	qMo3.2	PR12	1	3	17.20	17.37	7.1	6.7	0.18	0.05
	qMo3.3	IR12	2	3	26.70	26.83	6.4	7.2	0.67	0.05
**Mo**	*qMo3.3*	PR12	2	3	26.76	26.78	6.0	6.3	0.17	0.06
	qMo5.1	IR13	1	5	5.30	5.36	6.8	6.9	0.49	0.07
	qMo6.1	PR12	1	6	3.25	3.34	6.3	7.5	0.13	0.05
	qMo8.1	PR13	2	8	0.00	0.25	9.2	10.1	0.12	0.85
	qMo8.1	PR12	2	8	0.00	0.33	8.0	9.9	0.07	0.38
	qMo9.1	PR12	1	9	18.78	18.89	5.2	9.5	0.09	0.91
	qMo10.1	IR13	1	10	4.66	4.76	7.8	7.2	0.72	0.06
	qMo10.2	PR12	2	10	5.17	5.28	5.4	4.4	0.10	0.09
	qMo10.2	IR13	2	10	5.17	5.36	7.1	7.5	0.59	0.09
	qMo10.3	PR13	1	10	16.29	16.43	5.8	5.3	0.12	0.07
	qMo10.4	PR13	1	10	16.84	16.99	5.1	4.1	0.09	0.07
	qMo11.1	PR13	1	11	0.69	0.72	5.2	4.6	0.06	0.19
	qMo12.1	PR12	1	12	4.53	4.61	5.7	7.1	0.10	0.05
	qMo12.2	PR12	1	12	4.73	4.79	5.5	6.3	0.16	0.05
	qMo12.3	PR13	1	12	10.24	10.24	6.1	6.6	0.18	0.07
	qMo12.4	PR13	1	12	10.98	11.06	5.5	7.8	0.15	0.05
	qMo12.5	PR13	1	12	11.13	11.56	7.9	9.6	0.20	0.05
	qMo12.6	PR13	1	12	12.11	12.26	7.0	8.0	0.14	0.06
	qMo12.7	PR13	1	12	12.36	12.98	6.8	7.9	0.13	0.07
	qMo12.8	IR13	1	12	13.85	13.88	6.1	5.3	0.59	0.05
	qMo12.9	IR13	1	12	14.15	14.16	6.1	7.3	0.64	0.10
**Na**	*qNa1.1*	PR12	1	1	7.78	8.22	7.1	5.9	4.87	0.05
	qNa1.2	IR13	2	1	11.01	11.54	8.9	9.7	10.28	0.27
	qNa1.2	IR12	2	1	11.46	11.52	6.3	5.2	2.75	0.77
	qNa1.3	PR13	1	1	22.46	22.49	5.2	6.1	3.99	0.16
	qNa2.1	PR12	1	2	3.19	3.33	5.8	6.3	4.88	0.06
	qNa2.2	PR13	1	2	19.69	19.71	5.5	8.0	5.17	0.07
	qNa2.3	IR12	1	2	22.47	22.54	5.9	4.9	2.34	0.26
	qNa2.4	PR12	1	2	35.37	35.42	6.3	6.1	4.74	0.07
	qNa3.1	IR12	1	3	26.16	26.18	5.6	5.8	1.19	0.68
	qNa5.1	PR13	1	5	7.14	7.23	5.2	4.4	4.97	0.05
	qNa5.2	PR13	1	5	16.59	16.73	5.8	5.2	3.35	0.14
	qNa8.1	PR13	1	8	1.71	1.71	6.0	7.8	3.55	0.26
	qNa8.2	PR12	1	8	20.67	20.72	8.5	6.9	4.48	0.08
	qNa9.1	PR13	1	9	0.41	1.16	9.4	9.8	7.20	0.06
	qNa9.2	PR13	1	9	20.58	20.71	5.3	7.1	3.80	0.16
	qNa11.1	PR13	1	11	16.71	16.94	6.2	5.4	4.76	0.05
	qNa11.2	PR12	1	11	25.52	25.52	7.3	5.8	2.76	0.13
	qNa11.3	PR13	1	11	26.52	26.53	6.2	6.5	5.83	0.06
	qNa11.4	PR13	1	11	26.96	27.19	6.2	6.0	4.94	0.09

Trait	QTL	Env	No. env	Chr	Start Mb	End Mb	-log$_{10}$(P)	PVE (%)	Add. Effect	High All. Freq.
**Na**	*qNa11.5*	PR13	2	11	27.37	27.48	6.2	5.4	5.54	0.05
	qNa11.5	IR13	2	11	27.68	27.82	5.2	4.8	10.73	0.16
**P**	*qP1.1*	IR12	1	1	27.25	27.38	5.6	4.8	527.96	0.06
	qP6.1	PR13	1	6	6.72	6.74	5.2	4.7	272.37	0.82
	qP6.2	IR13	1	6	11.83	11.99	5.3	5.5	321.93	0.58
	qP6.3	IR13	1	6	12.26	12.29	5.9	9.2	459.57	0.71
**Zn**	*qZn1.1*	PR13	1	1	18.29	18.30	5.4	4.5	4.28	0.07
	qZn1.2	IR12	1	1	38.28	38.43	6.0	9.1	2.86	0.86
	qZn2.1	PR13	1	2	24.85	24.97	5.6	5.2	1.51	0.73
	qZn2.2	IR13	1	2	25.51	25.62	5.1	5.7	2.03	0.63
	qZn3.1	PR12	1	3	6.34	6.45	5.5	6.2	1.91	0.54
	qZn3.2	PR12	1	3	6.65	6.79	6.2	6.5	2.12	0.36
	qZn6.1	IR13	1	6	29.54	29.56	5.5	5.6	5.40	0.08
	qZn7.1	IR13	1	7	3.28	3.31	5.8	5.7	4.16	0.12
	qZn7.2	**IR13**	**4**	**7**	**29.26**	**29.43**	6.3	5.0	4.88	0.11
	qZn7.2	**PR13**	**4**	**7**	**29.26**	**29.33**	6.4	5.1	3.87	0.16
	qZn7.2	**PR12**	**4**	**7**	**29.26**	**29.42**	6.9	8.0	2.94	0.19
	qZn7.2	**IR12**	**4**	**7**	**29.26**	**29.41**	6.1	5.2	7.34	0.06
	qZn7.3	IR12	3	7	29.42	29.67	6.5	5.4	6.38	0.08
	qZn7.3	IR13	3	7	29.47	29.67	5.5	4.3	4.04	0.12
	qZn7.3	PR12	3	7	29.52	29.67	5.4	5.0	4.00	0.07
	qZn9.1	PR13	1	9	0.66	0.78	5.4	4.2	2.47	0.20
	qZn11.1	PR12	1	11	5.70	5.71	5.1	6.5	2.00	0.34
	qZn12.1	PR13	1	12	9.76	9.76	5.3	4.3	2.74	0.06

Table S4: Summary of the QTL for the agronomic traits including days to flowering (DF, days), grain yield (GY, kg ha^{-1}), plant height (PH, cm), and thousand grain weight (TGW, g) of the PRAY panel in four environments (IR12: IRRI 2012, IR13: IRRI 2013, PR12: PhilRice 2012, PR13: PhilRice 2013) detected by mixed linear models. QTL detected in more than one environment are shaded. Chr, chromosome; Start/End: physical position of the linkage block; Add. Effect: estimated additive effect, PVE: phenotypic variation explained by the QTL (%). High All. Freq: Frequency of the higher value allele.

Trait	QTL	Env	No. env	Chr	Start Mb	End Mb	$-log_{10}(P)$	PVE (%)	Add. Effect	High All. Freq.
**DF**	*qDF1.1*	IR12	1	1	5.64	5.66	5.9	4.8	16.89	0.89
	qDF1.1	IR13	1	1	5.84	6.09	6.6	10.4	2.51	0.13
	qDF1.2	IR13	1	1	12.59	12.61	5.1	4.3	13.75	0.08
	qDF2.1	PR12	1	2	1.51	1.52	5.3	4.3	8.71	0.11
	qDF2.2	PR13	1	2	34.44	34.45	7.9	14.7	9.73	0.35
	qDF6.1	IR13	2	6	17.34	17.41	5.7	5.2	18.76	0.06
	qDF6.2	IR13	2	6	17.72	17.85	5.5	6.0	19.25	0.05
	qDF6.3	IR13	1	6	22.53	22.54	5.9	5.2	16.94	0.05
	qDF6.4	IR13	1	6	25.10	25.29	5.2	4.1	16.30	0.06
	qDF7.1	IR13	1	7	3.41	3.58	5.7	6.9	10.23	0.65
**GY**	*qGY1.1*	IR13	1	1	4.01	4.03	6.0	7.1	75.66	0.18
	qGY2.1	PR13	1	2	1.10	1.14	5.3	6.6	99.93	0.55
	qGY9.1	PR13	1	9	6.96	7.09	6.4	6.7	250.87	0.05
	qGY10.1	IR13	1	10	16.81	17.20	5.8	5.1	168.70	0.95
	qGY12.1	PR13	1	12	21.87	21.92	5.3	7.2	59.52	0.44
	qGY12.2	PR13	1	12	22.03	22.05	5.3	7.2	150.26	0.08
	qGY12.3	PR13	1	12	24.75	24.78	6.1	6.5	137.23	0.16
**PH**	*qPH1.1*	IR12	2	1	27.25	27.29	6.1	3.0	28.79	0.92
	qPH1.1	PR13	2	1	27.25	27.29	7.8	3.9	19.20	0.92
	qPH1.2	IR12	2	1	32.69	32.72	5.2	3.1	36.50	0.74
	qPH1.2	PR13	2	1	32.69	32.81	5.2	3.2	30.07	0.74
	qPH1.3	PR13	1	1	33.17	33.33	5.7	3.7	30.51	0.50
	qPH1.4	PR13	2	1	33.81	34.01	5.5	4.0	28.91	0.46
	qPH1.4	IR13	2	1	33.91	33.91	5.5	4.1	32.05	0.40
	qPH1.5	PR13	1	1	34.07	34.40	6.6	5.4	28.88	0.43
	qPH1.6	IR13	1	1	34.20	34.39	5.4	3.8	45.49	0.81
	qPH1.7	IR13	1	1	35.13	35.21	6.4	4.8	44.53	0.77
	qPH1.8	IR13	1	1	35.53	35.72	5.4	3.6	45.67	0.80
	qPH1.9	IR13	1	1	36.60	36.66	5.2	3.9	9.30	0.08
	qPH1.10	IR12	3	1	37.75	37.96	6.6	6.8	52.34	0.75
	qPH1.10	IR13	3	1	37.75	37.96	9.0	10.0	49.39	0.75
	qPH1.10	PR13	3	1	37.75	38.38	8.6	9.3	43.06	0.76
	qPH1.11	IR13	2	1	38.09	38.11	8.2	8.2	48.85	0.74
	qPH1.11	IR12	2	1	38.15	38.32	5.5	4.9	53.31	0.70
	qPH1.12	IR13	3	1	38.22	38.56	8.7	12.8	49.28	0.69
	qPH1.12	PR13	3	1	38.39	38.63	7.3	10.6	41.80	0.70
	qPH1.12	IR12	3	1	38.48	38.56	6.2	8.1	50.61	0.69

Trait	QTL	Env	No. env	Chr	Start Mb	End Mb	$-log_{10}(P)$	PVE (%)	Add. Effect	High All. Freq.
PH	*qPH1.13*	IR12	3	1	39.00	39.07	5.9	6.7	54.09	0.71
	qPH1.13	IR13	3	1	38.85	39.29	9.7	11.8	50.28	0.71
	qPH1.13	PR13	3	1	38.85	39.29	9.6	11.6	43.17	0.71
	qPH1.14	IR13	2	1	39.45	39.62	6.6	8.6	51.62	0.71
	qPH1.14	PR13	2	1	39.45	39.55	6.0	7.8	43.84	0.72
	qPH1.15	IR13	1	1	39.73	39.84	5.6	5.5	46.29	0.69
	qPH1.16	PR13	1	1	41.14	41.18	5.2	2.9	7.92	0.81
	qPH1.15	PR12	1	1	41.51	41.71	8.0	7.7	19.00	0.91
	qPH3.1	IR12	1	3	0.21	0.50	7.6	5.0	23.30	0.59
	qPH4.1	PR12	1	4	18.82	18.95	5.8	4.9	12.37	0.83
	qPH4.2	PR12	1	4	19.24	19.27	5.2	4.4	18.07	0.85
	qPH5.1	IR12	1	5	14.03	14.03	6.1	3.2	30.99	0.94
	qPH6.1	PR12	1	6	27.86	27.94	5.7	5.0	9.90	0.26
	qPH8.1	IR12	1	8	1.30	1.31	6.4	4.1	20.41	0.43
	qPH8.2	PR13	1	8	20.23	20.31	6.8	5.2	30.04	0.49
	qPH9.1	PR12	1	9	2.91	3.01	5.1	4.8	13.90	0.81
	qPH11.1	IR12	1	11	0.52	0.66	5.2	4.1	0.94	0.93
	qPH11.2	IR12	1	11	23.01	23.17	6.1	3.5	34.04	0.91
	qPH12.1	IR12	1	12	26.01	26.14	5.9	3.6	15.14	0.09
TGW	*qTGW2.1*	IR13	1	2	27.64	27.76	5.3	5.5	1.71	0.86
	qTGW3.1	PR13	1	3	16.73	16.92	5.2	6.0	1.43	0.74
	qTGW4.1	IR13	2	4	0.42	0.51	5.2	5.4	1.95	0.13
	qTGW4.1	PR13	2	4	0.42	0.51	5.4	5.5	2.37	0.08
	qTGW5.1	IR13	1	5	5.36	5.38	6.1	7.5	1.32	0.37
	qTGW6.1	IR13	2	6	22.54	22.54	6.2	6.6	3.68	0.94
	qTGW6.1	PR13	2	6	22.54	22.54	5.6	5.7	3.29	0.94
	qTGW7.1	PR13	1	7	20.20	20.22	6.0	6.7	1.38	0.86
	qTGW7.2	PR13	1	7	22.16	22.28	5.5	4.6	3.18	0.95
	qTGW7.3	IR13	2	7	22.37	22.68	8.3	7.5	3.68	0.95
	qTGW7.3	PR13	2	7	22.37	23.55	7.7	7.4	3.34	0.94
	qTGW7.4	IR13	1	7	22.74	23.51	7.6	7.7	4.32	0.92
	qTGW9.1	PR13	1	9	12.60	12.62	5.6	5.9	1.47	0.40
	qTGW12.1	IR13	2	12	7.71	7.75	5.6	6.4	1.19	0.37
	qTGW12.1	PR13	2	12	7.71	7.75	5.8	6.6	1.17	0.37
	qTGW12.2	IR13	1	12	7.88	7.88	5.1	7.0	1.09	0.38

Table S5: Genes included in the localized region delimited by the most significantly associated SNPs with element concentrations. For each gene locus are listed the information relative to the start and stop position (bp), and annotation (information from https://rapdb.dna.affrc.go.jp/download/irgsp1.html). Transporters are coded in yellow, flowering genes in pink, starch synthesis in orange

**GeneID**	**Chr**	**Location(bp)**	**Description**	**Linked QTL**	**QTL within 300kb**
Os03g0386700	3	15395285-15397801	*OsSCP12* Serine carboxypeptidase family		*qB3.1, qCa3.1, qCo3.1, qMn3.1, qMo3.2, qCa3.2, qTGW3.1*
Os03g0386800	3	15400468-15403841	*OsSCP13* Serine carboxypeptidase family		
Os03g0388100	3	15479861-15482139	ATPase, P-type, K/Mg/Cd/Cu/Zn/Na/Ca/Na/H-transporter		
Os03g0389000	3	15563469-15564927	Metacaspase 3; Serine-type carboxypeptidase		
Os03g0389100	3	15567899-15572135	Metacaspase 8; Serine-type carboxypeptidase		
Os03g0389400	3	15584826-15585200	Metacaspase 2; Serine-type carboxypeptidase		
Os03g0389501	3	15584826-15587152	Metacaspase 2; Serine-type carboxypeptidase		
Os03g0392600	3	15763402-15767313	*OsSCP14* - Serine Carboxypeptidase homologue	*qMo3.2*	
Os03g0393100	3	15795756-15796260	Petinoid-inducible serine carboxypeptidase.		
Os03g0393300	3	15789733-15796658	*OsSCP16* - Serine Carboxypeptidase homologue *GS5*		
Os03g0393375	3	15801286-15801911	*OsSCP17* - Serine Carboxypeptidase homologue		
Os03g0393700	3	15823663-15829107	*OsSCP18* - Serine Carboxypeptidase homologue		
Os03g0395000	3	15923873-15927434	Stroma-localized heme oxygenase 2	*qCa3.1*	
Os03g0395100	3	15932524-15936871	Protein phosphatase 2C domain.		
Os03g0397300	3	16051260-16058325	Metallophosphoesterase domain.		
Os03g0397400	3	16061151-16065169	Low affinity calcium transporter CAX2		
Os03g0397600	3	16071005-16072780	Glycoside hydrolase, family 17 protein		
Os03g0398250	3	16117257-16117934	Zinc finger, RING/FYVE/PHD-type domain.		
Os03g0399000	3	16151224-16153558	Pectin methylesterase 12; cell wall modification		
Os03g0399532	3	16182022-16192749	Phosphatidylinositol-4-phosphate 5-kinase family.		
Os03g0400700	3	16250621-16252717	Late embryogenesis abundant protein 11		
Os03g0401366	3	16301277-16307632	***SUS1*; Sucrose synthase (EC 2.4.1.13)**	*qCo3.1*	
Os03g0406100	3	16645316-16646422	SPX domain-, Negative regulation of phosphate signaling		
Os03g0407400	3	16729501-16735109	GS3, Regulator of grain size and organ size	*qCa3.2, qTGW3.1*	
Os03g0410100	3	16888876-16892201	SUMO protease protein		
Os03g0411800	3	17007955-17011263	**Zinc transporter 2 (*ZIP2*)**		
Os03g0412300	3	17046919-17049240	Heavy metal transport/detoxification protein domai		
Os03g0412800	3	17067788-17072249	Glucose-6-phosphate dehydrogenase precursor.		
Os03g0413400	3	17109125-17113771	Glycosyl transferase, family 8 protein.		
Os03g0416300	3	17261821-17266546	Phytochelatin synthetase, brittle culm-like 2		
Os03g0417700	3	17340415-17342284	*GS3*. Phytochrome P450; Promotion of grain growth		

GeneID	Chr	Location(bp)	Description	Linked QTL	QTL within 300kb
Os06g0126100	6	1398084-1401474	ABC transporter		*qMn6.1 qB6.1 qK6.1*
Os06g0126250	6	1397921-1400968	High-affinity potassium transporters *OsHKT9*		
Os06g0127600	6	1459668-1465202	Glutathione transporter.		
Os06g0127700	6	1459192-1464339	Oligopeptide transporter 9.		
Os06g0129400	6	1554861-1561163	Vacuolar phosphate efflux transporter, OsSPX-MFS3	*qB6.1*	
Os06g0130400	6	1629717-1633629	ACC synthase; Ethylene biosynthesis, starch in endosperm	*qK6.1*	
Os06g0131500	6	1691885-1694140	Glucan endo-1, 3-beta-glucosidase 7; Glycoside hydrolase		
Os06g0131700	6	1699371-1702859	No apical meristem (NAM) protein		
Os06g0133000	6	1765622-1770656	**Granule-bound starch synthase 1 (*OsGBSS1*)**		
Os07g0686300	7	29152543-29153434	Zinc finger, RING/FYVE/PHD-type domain.		*qCo7.1 qZn7.2 qCu7.1*
Os07g0687200	7	29197727-29200392	CAM7 (CALMODULIN 7); calcium ion binding.		
Os07g0687900	7	29220283-29221843	Galactinol synthase, induce by water stress, salt stress		
Os07g0688000	7	29225276-29228504	Metallophosphoesterase domain.	*qCo7.1 qZn7.2*	
Os07g0689600	7	29323094-29324723	***OsNAS3* Nicotianamine synthase 3**		
Os07g0690300	7	29353044-29357315	Zinc finger, RING/FYVE/PHD-type domain.	*qZn7.2*	
Os07g0690800	7	29376261-29380972	Phytochelatin synthase 12		
Os07g0690900	7	29382724-29385096	Glycosyl-phosphatidyl inositol-anchored, brittle culm-like 8		
Os07g0691100	7	29388041-29390114	Pectin methylesterase 6.		
Os07g0692900	7	29478758-29483659	Ubiquitin-activating enzyme E1.	*qZn7.2 qCu7.1*	
Os07g0693100	7	29490011-29492173	Pyruvate decarboxylase isozyme 3 (EC 4.1.1.1)		
Os07g0694000	7	29545825-29548981	Phosphoinositide phospholipase C, Salt tolerance		
Os07g0694700	7	29583717-29586497	Ascorbate peroxidase, Carbohydrate metabolism		
Os07g0695100	7	29616705-29629223	Heading response regulator; Long-day repression		
Os08g0407200	8	19463926-19469347	Trypsin-like serine and cysteine proteases, grain yield		*qCo8.2, qMg8.1, qNa8.1, qNa8.2, qPH8.2*
Os08g0409100	8	19578770-19583372	Trehalose-6-phosphate phosphatase.		
Os08g0410500	8	19647746-19649747	Carbohydrate transporter/ sugar porter/ transporter.	*qMg8.1, qPH8.2*	
Os08g0414700	8	19830328-19836087	Trehalose-6-phosphate synthase.	*qPH8.2*	
Os08g0421700	8	20184791-20186177	Zinc finger, CCHC-type domain.	*qCo8.2*	
Os08g0422200	8	20219069-20221985	**Cation efflux family; OsMTP12 Metal tolerance protein**	*qPH8.2*	
Os08g0423500	8	20283045-20285487	Carbonic anhydrase, CAH1-like domain.		
Os08g0423600	8	20312582-20314716	Carbonic anhydrase		
Os08g0425300	8	20429460-20431037	Endoglucanase 21.		
Os08g0430500	8	20812503-20815632	14-3-3 protein, Florigen receptor, Flowering		

Table S6: Detailed information describing the growing conditions of the four environments (IR12: IRRI 2012, IR13: IRRI 2013, PR12: PhilRice 2012, PR13: PhilRice 2013) The trials were planted in three replicates with roup block design. Genotypes within groups were randomised in the field. DAS: days after sowing, w: width, l: length

	**IR12**	**IR13**	**PR12**	**PR13**
**Location**	14°15'N 121°27'E	14°15'N 121°27'E	15°67'N 120°89'E	15°67'N 120°89'E
**Sowing date**	12/jun/2012	03/jan/2013	09/jan/2012	21/12/2012
**Tem mean**	24-27 °C	24-28 °C	24-27 °C	23-28 °C
**Temp high**	27-31°C	27-33 °C	29-33 °C	29-34 °C
**Temp low**	22-24 °C	22-25 °C	20-23 °C	19-23 °C
**Rainfall**	11-236 mm	17-147 mm	29-248 mm	5-147 mm
**Humidity**	82-88 %	74-83 %	76-87 %	74-83%
**Sun hours**	167-300 h	152-369 h	143-351 h	140-349 h
**Planting method**	Transplanted, 16-day old seedlings	Transplanted, 14-day old seedlings	Transplanted, 18-day old seedlings	Transplanted, 22-day old seedlings
**Planting design**	Genotypes were grouped based on days to flowering	Genotypes were grouped based on days to flowering and plant height.	Genotypes were grouped based on days to flowering	Genotypes were grouped based on days to flowering and plant height.
**Block w × l**	0.6 m × 3m	1.2 m × 1 m	0.2 m × 3 m	0.6 m × 3 m
**Watering**	Irrigation starting at 7-10 days after transplanting; maintained 3-5 cm until panicle initiation; reduce to very shallow water layer before each N application; re-irrigate within 1-2 days after N application; Standing water was maintained from flowering and drained one week before harvest.	15 cm of continuous lamina until flowering, then decrease to 5 cm	Irrigation starting at 7-10 days after transplanting maintained 3-5 cm until panicle initiation; reduce to very shallow water layer before each N application; re-irrigate within 1-2 days after N application; Standing water was maintained from flowering and drained one week before harvest.	Irrigation starting at 7-10 days after transplanting maintained 3-5 cm until panicle initiation; reduce to very shallow water layer before each N application; re-irrigate within 1-2 days after N application; Standing water was maintained from flowering and drained one week before harvest.
**Fertiliser**	Total of 200 kg N per ha applied in four splits (basal, midtillering, panicle initiation and at flowering).<br><br>30 kg P and 40 kg K were applied at transplanting.	Total of 180 kg N per ha applied in four splits (basal, midtillering, panicle initiation and at flowering).<br><br>30 kg P and 40 kg K were applied at transplanting.	Nitrogen was applied in three splits: 60 kg N/ha as basal as complete fertilizer, 40 kg N/ha as urea at 40 DAS and 60 kg N/ha as urea at 60 DAS.<br><br>60 kg P and 60 kg K per hectare were applied at basal.<br>Zinc was applied as zinc sulfate at 5 kg/ha at basal.	Nitrogen was applied in two splits: 60 kg N/ha at 12 DAS as complete fertilizer and at 52 DAS as urea. 60 kg P and 60 kg K per hectare were applied at 12 DAS.<br><br>Zinc was applied as zinc sulfate at 5 kg Zn/ha at basal.

Fig S1: Manhattan plots (left) and QQ plots (right) showing results of association analysis for grain element concentrations and agronomic traits. The orange line indicates a significance threshold of $-\log_{10}(p) > 5.12$**.**

Boron (B)

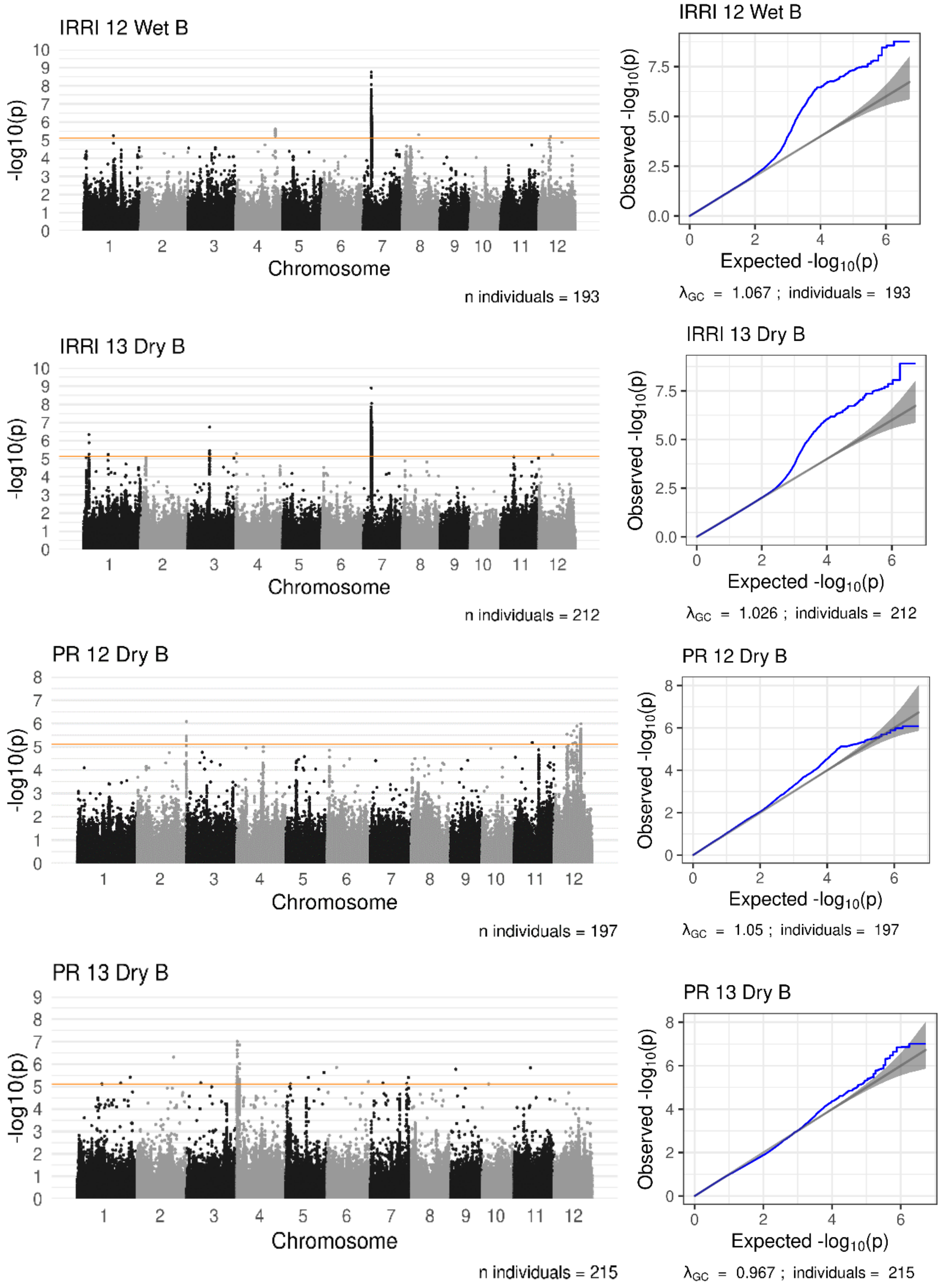

Calcium (Ca)

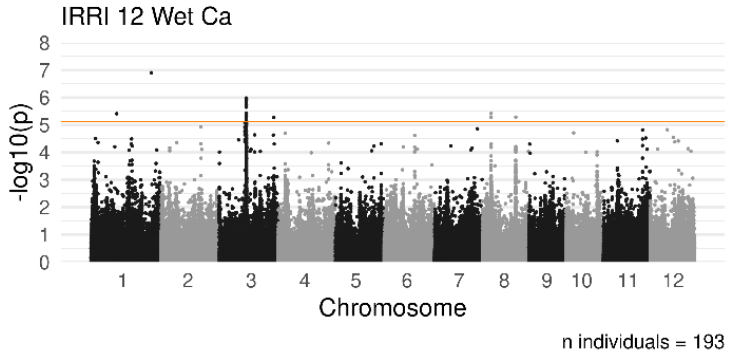
IRRI 12 Wet Ca
-log10(p)
Chromosome
n individuals = 193

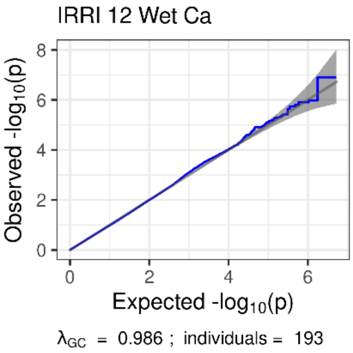
IRRI 12 Wet Ca
Observed $-\log_{10}(p)$
Expected $-\log_{10}(p)$
λ_{GC} = 0.986 ; individuals = 193

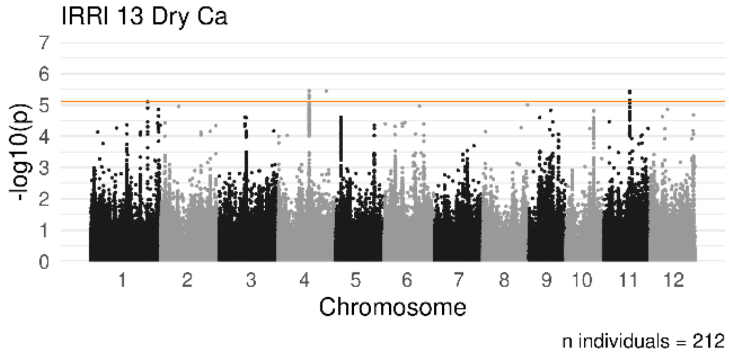
IRRI 13 Dry Ca
-log10(p)
Chromosome
n individuals = 212

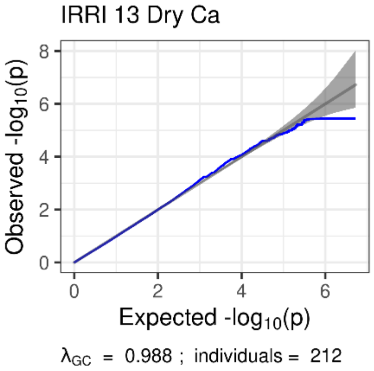
IRRI 13 Dry Ca
Observed $-\log_{10}(p)$
Expected $-\log_{10}(p)$
λ_{GC} = 0.988 ; individuals = 212

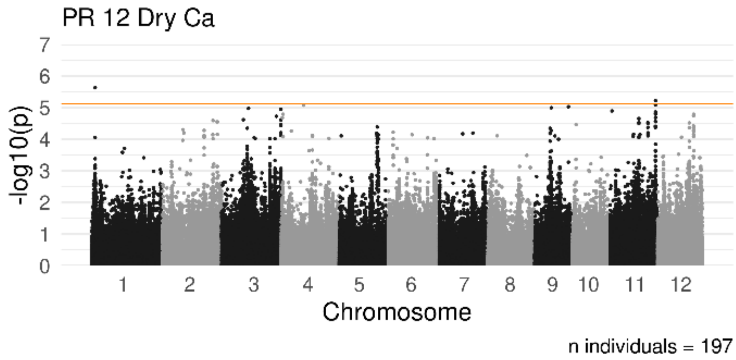
PR 12 Dry Ca
-log10(p)
Chromosome
n individuals = 197

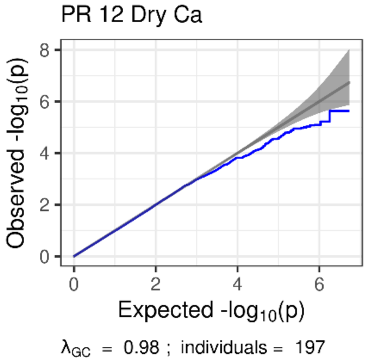
PR 12 Dry Ca
Observed $-\log_{10}(p)$
Expected $-\log_{10}(p)$
λ_{GC} = 0.98 ; individuals = 197

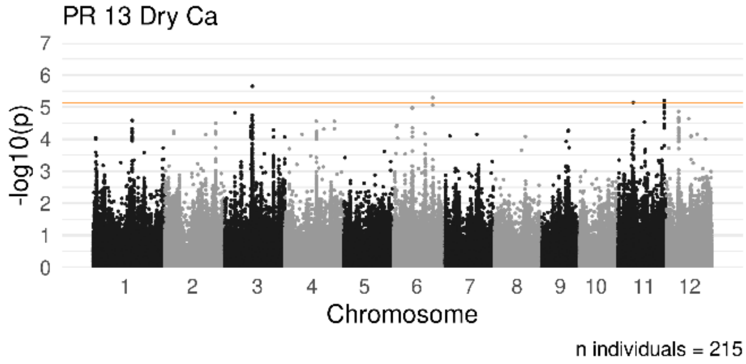
PR 13 Dry Ca
-log10(p)
Chromosome
n individuals = 215

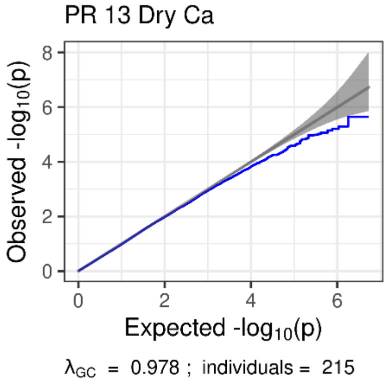
PR 13 Dry Ca
Observed $-\log_{10}(p)$
Expected $-\log_{10}(p)$
λ_{GC} = 0.978 ; individuals = 215

Cobalt (Co)

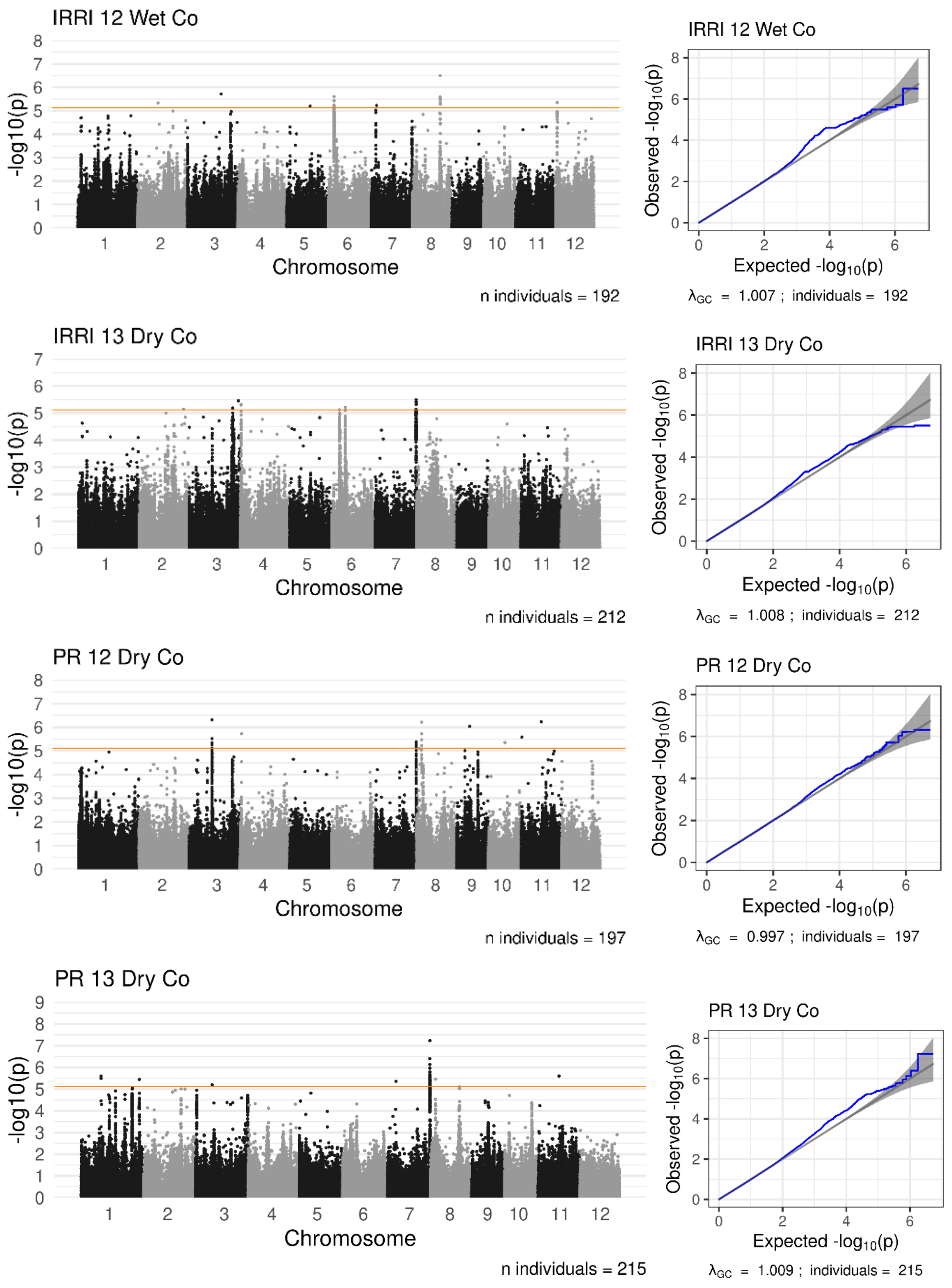
IRRI 12 Wet Co
-log10(p)
Chromosome
n individuals = 192
IRRI 12 Wet Co
Observed -log10(p)
Expected -log10(p)
λGC = 1.007 ; individuals = 192
IRRI 13 Dry Co
-log10(p)
Chromosome
n individuals = 212
IRRI 13 Dry Co
Observed -log10(p)
Expected -log10(p)
λGC = 1.008 ; individuals = 212
PR 12 Dry Co
-log10(p)
Chromosome
n individuals = 197
PR 12 Dry Co
Observed -log10(p)
Expected -log10(p)
λGC = 0.997 ; individuals = 197
PR 13 Dry Co
-log10(p)
Chromosome
n individuals = 215
PR 13 Dry Co
Observed -log10(p)
Expected -log10(p)
λGC = 1.009 ; individuals = 215

Copper (Cu)

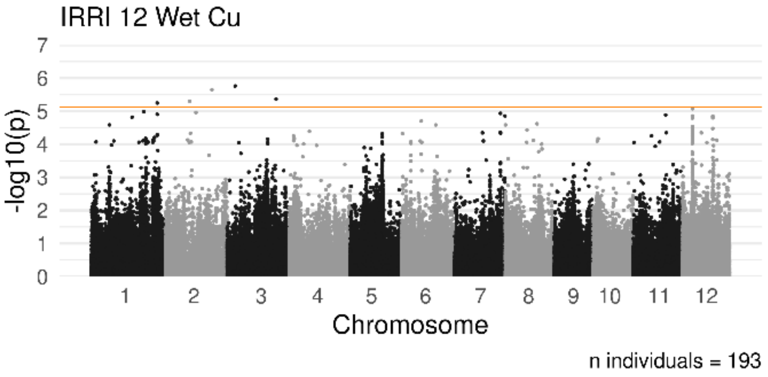
IRRI 12 Wet Cu
-log10(p)
Chromosome
n individuals = 193

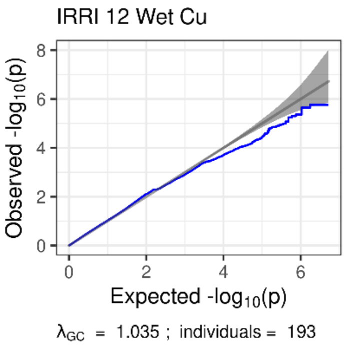
IRRI 12 Wet Cu
Observed -log10(p)
Expected -log10(p)
λGC = 1.035 ; individuals = 193

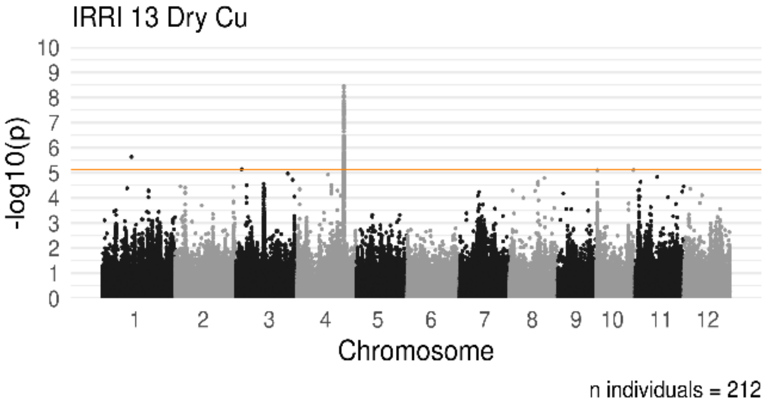
IRRI 13 Dry Cu
-log10(p)
Chromosome
n individuals = 212

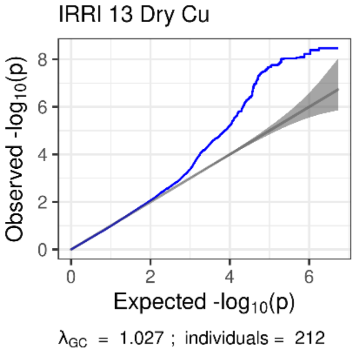
IRRI 13 Dry Cu
Observed -log10(p)
Expected -log10(p)
λGC = 1.027 ; individuals = 212

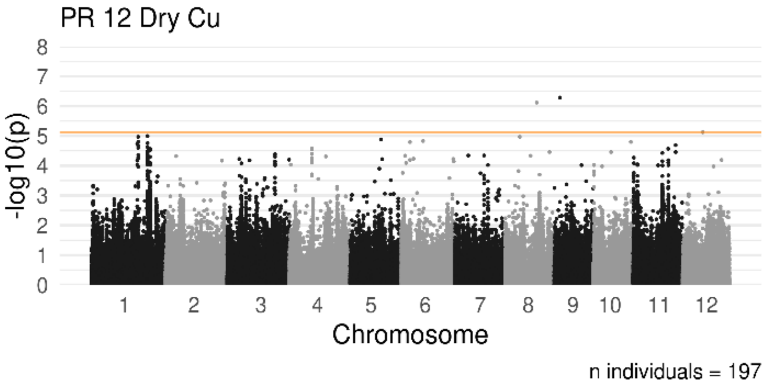
PR 12 Dry Cu
-log10(p)
Chromosome
n individuals = 197

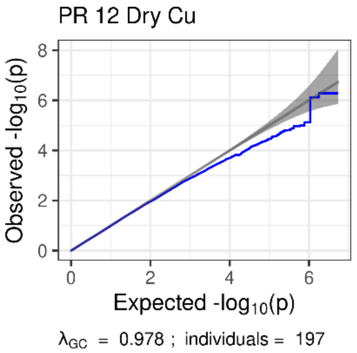
PR 12 Dry Cu
Observed -log10(p)
Expected -log10(p)
λGC = 0.978 ; individuals = 197

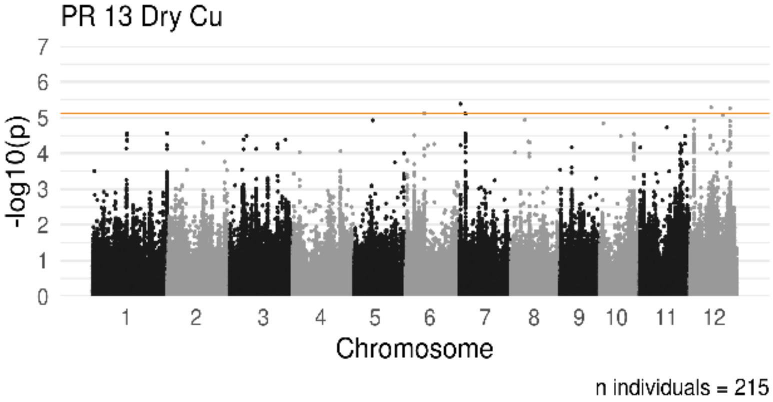
PR 13 Dry Cu
-log10(p)
Chromosome
n individuals = 215

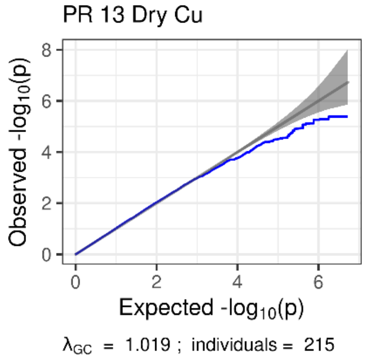
PR 13 Dry Cu
Observed -log10(p)
Expected -log10(p)
λGC = 1.019 ; individuals = 215

Iron (Fe)

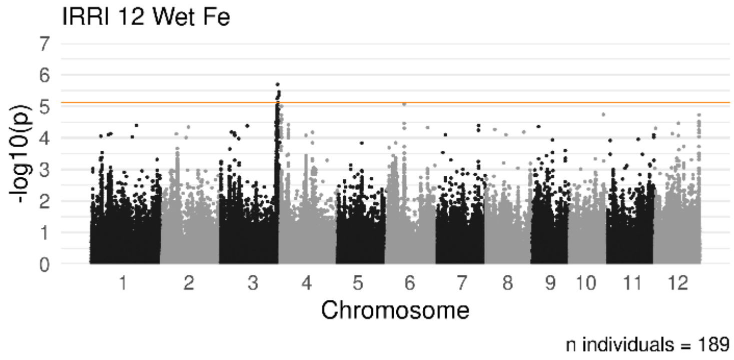
IRRI 12 Wet Fe
-log10(p)
Chromosome
1 2 3 4 5 6 7 8 9 10 11 12
n individuals = 189

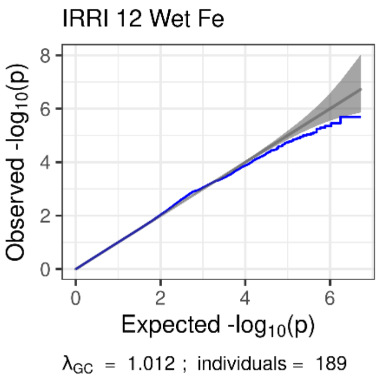
IRRI 12 Wet Fe
Observed $-\log_{10}(p)$
Expected $-\log_{10}(p)$
λ_{GC} = 1.012 ; individuals = 189

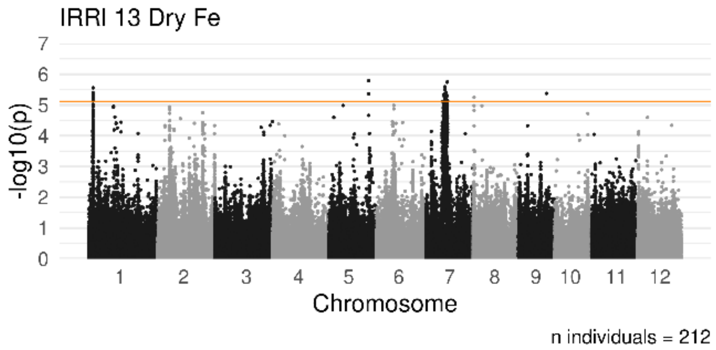
IRRI 13 Dry Fe
-log10(p)
Chromosome
1 2 3 4 5 6 7 8 9 10 11 12
n individuals = 212

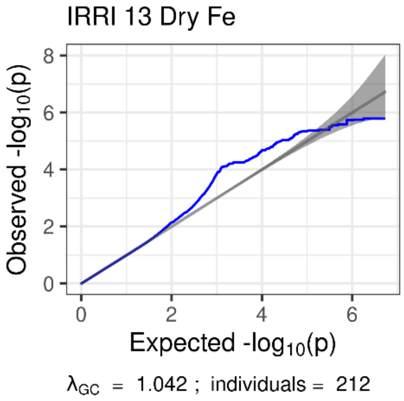
IRRI 13 Dry Fe
Observed $-\log_{10}(p)$
Expected $-\log_{10}(p)$
λ_{GC} = 1.042 ; individuals = 212

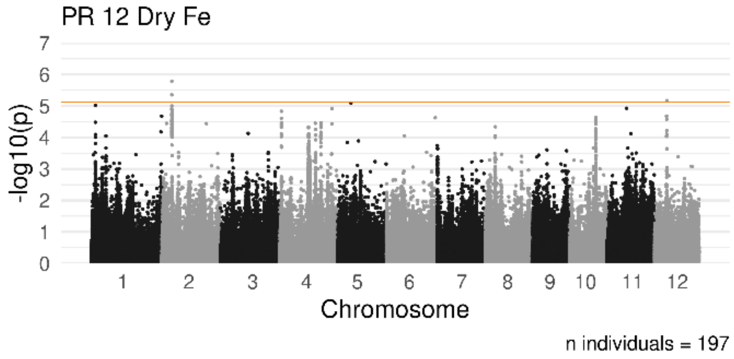
PR 12 Dry Fe
-log10(p)
Chromosome
1 2 3 4 5 6 7 8 9 10 11 12
n individuals = 197

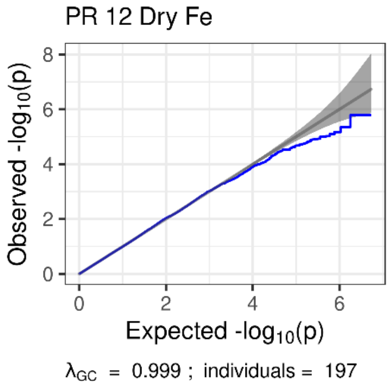
PR 12 Dry Fe
Observed $-\log_{10}(p)$
Expected $-\log_{10}(p)$
λ_{GC} = 0.999 ; individuals = 197

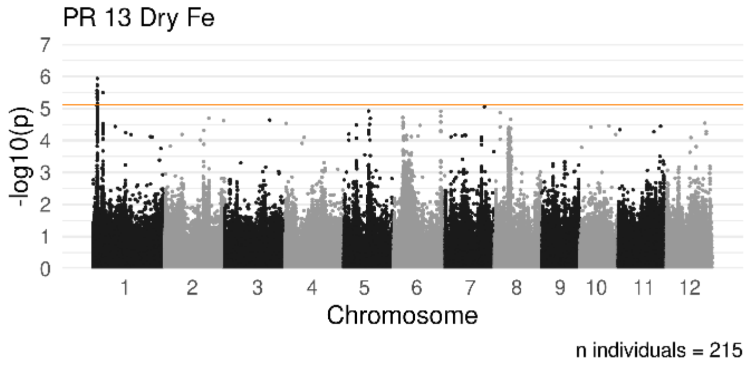
PR 13 Dry Fe
-log10(p)
Chromosome
1 2 3 4 5 6 7 8 9 10 11 12
n individuals = 215

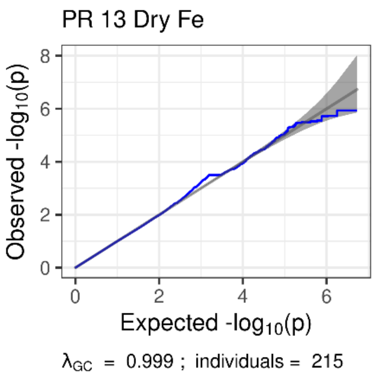
PR 13 Dry Fe
Observed $-\log_{10}(p)$
Expected $-\log_{10}(p)$
λ_{GC} = 0.999 ; individuals = 215

Potassium (K)

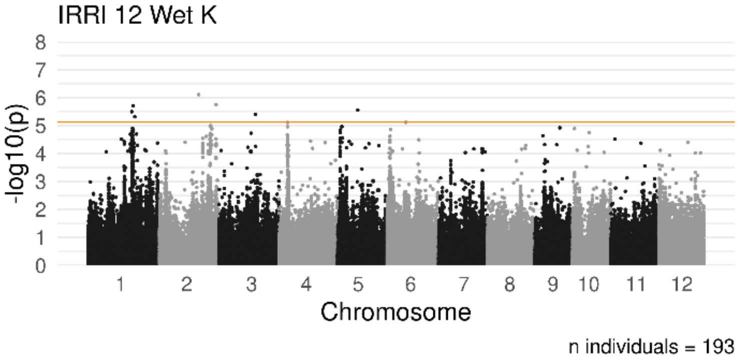
IRRI 12 Wet K
-log10(p)
Chromosome
n individuals = 193

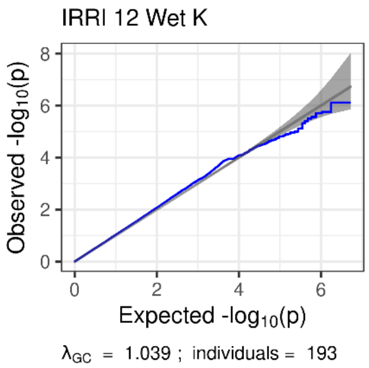
IRRI 12 Wet K
Observed -log10(p)
Expected -log10(p)
λGC = 1.039 ; individuals = 193

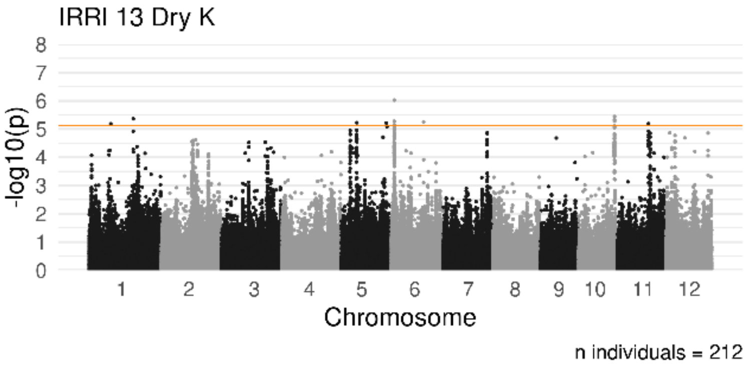
IRRI 13 Dry K
-log10(p)
Chromosome
n individuals = 212

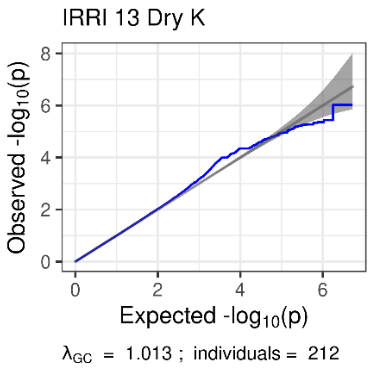
IRRI 13 Dry K
Observed -log10(p)
Expected -log10(p)
λGC = 1.013 ; individuals = 212

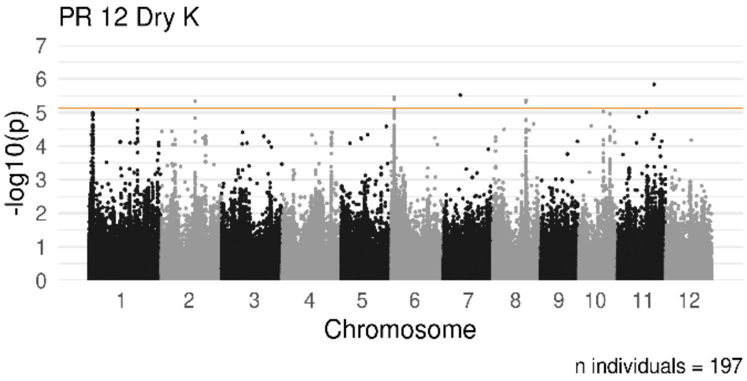
PR 12 Dry K
-log10(p)
Chromosome
n individuals = 197

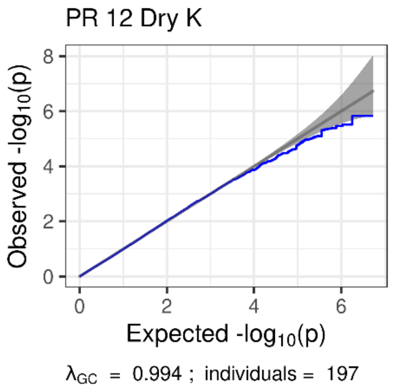
PR 12 Dry K
Observed -log10(p)
Expected -log10(p)
λGC = 0.994 ; individuals = 197

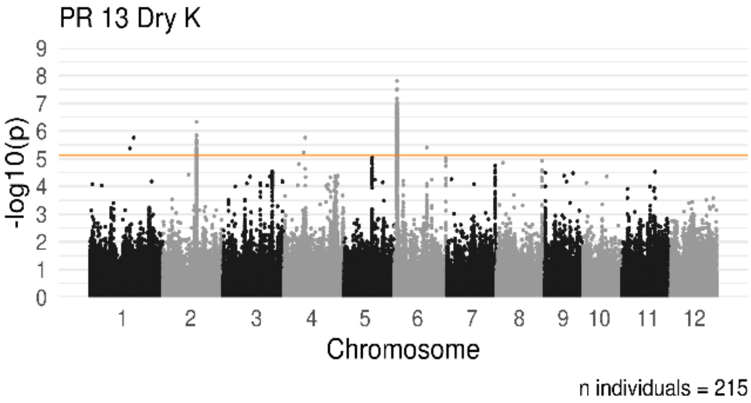
PR 13 Dry K
-log10(p)
Chromosome
n individuals = 215

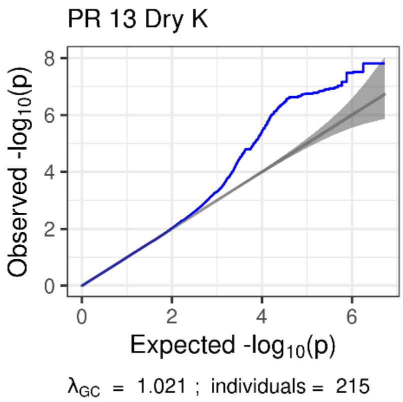
PR 13 Dry K
Observed -log10(p)
Expected -log10(p)
λGC = 1.021 ; individuals = 215

Magnesium (Mg)

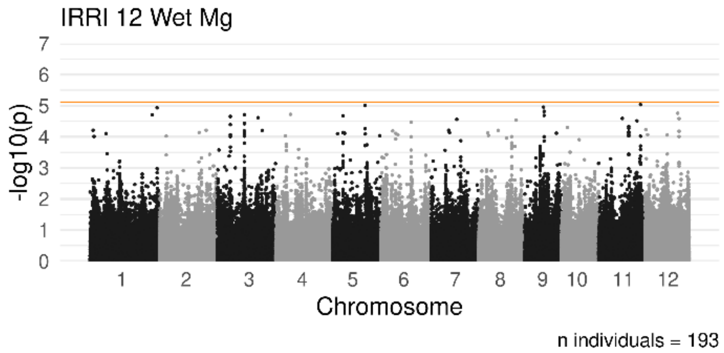

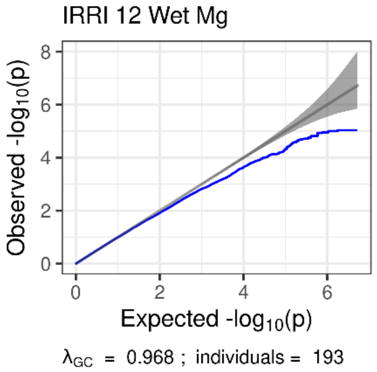

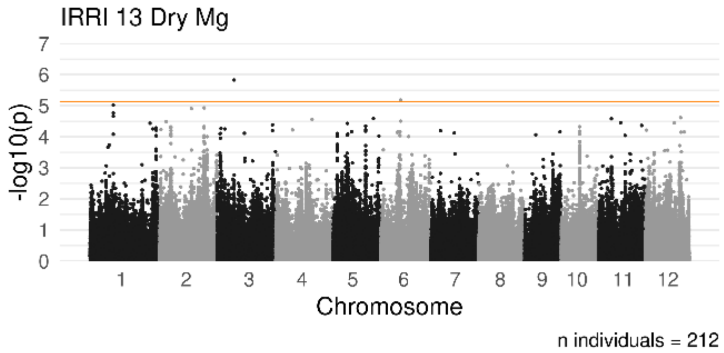

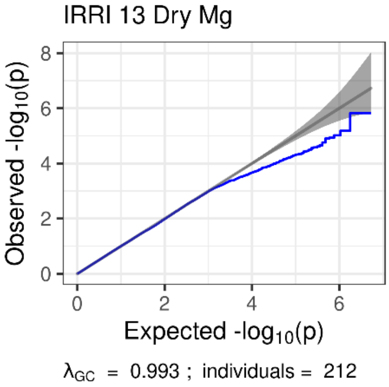

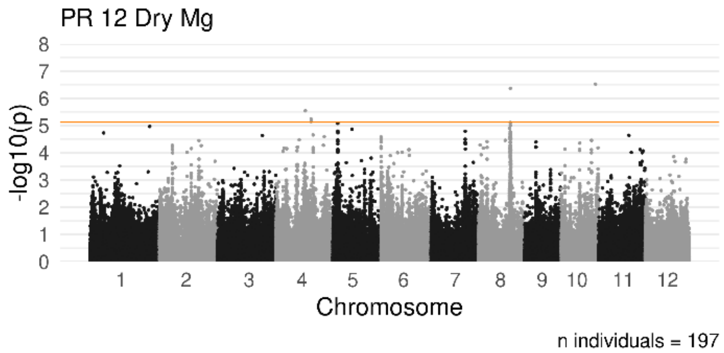

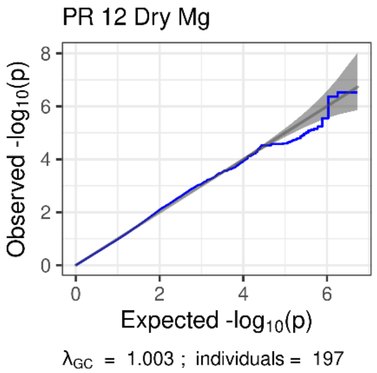

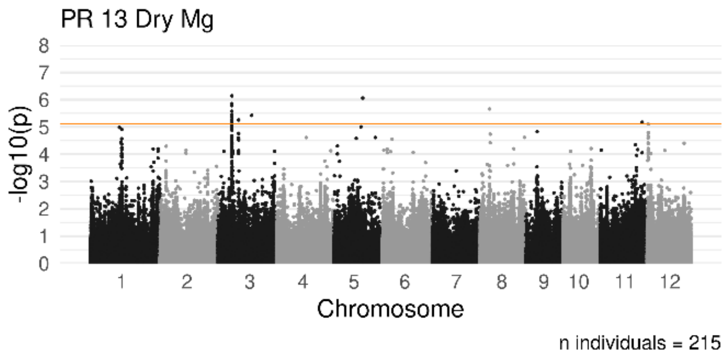

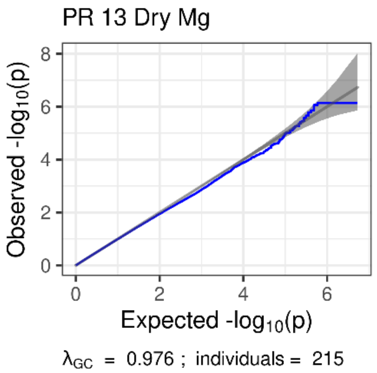

Manganese (Mn)

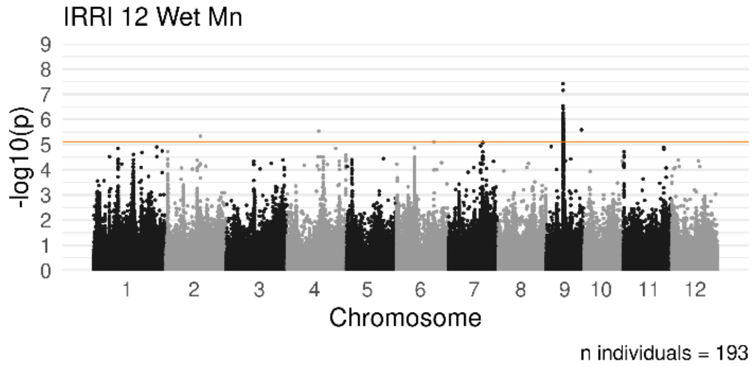

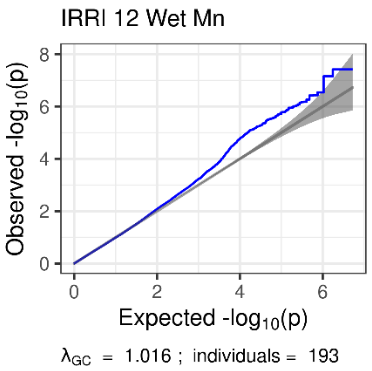

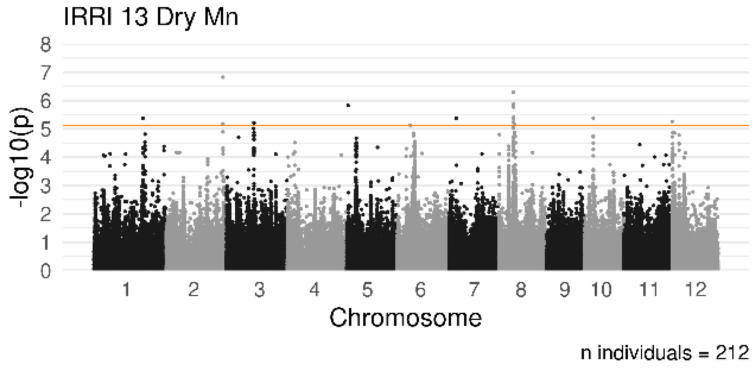

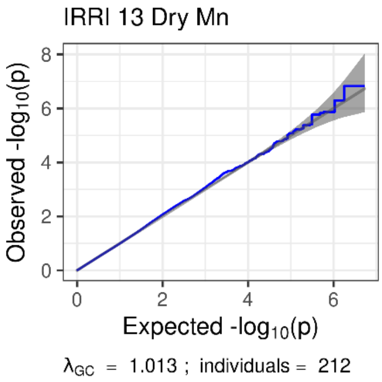

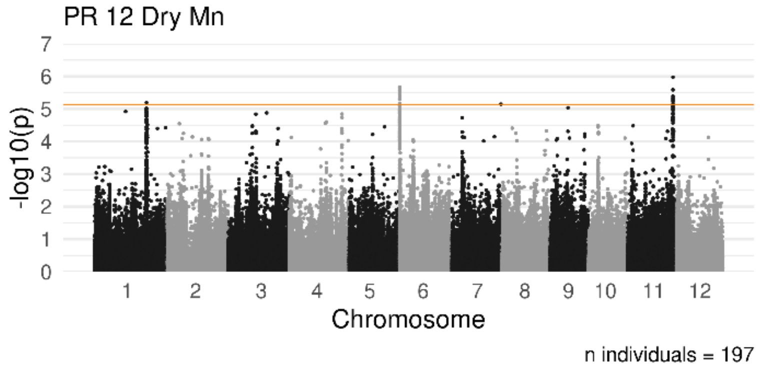

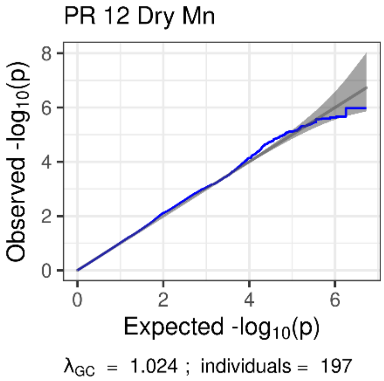

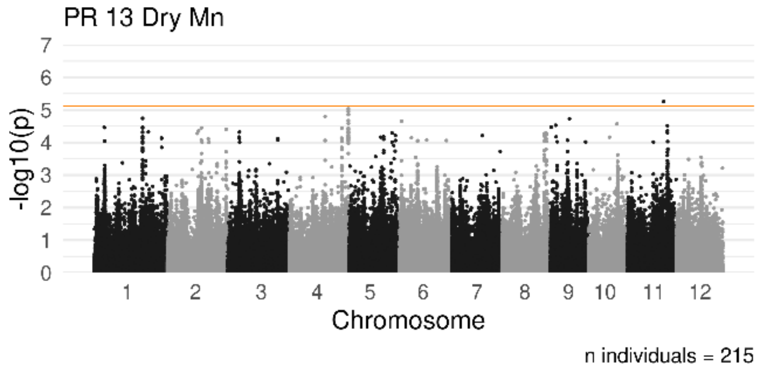

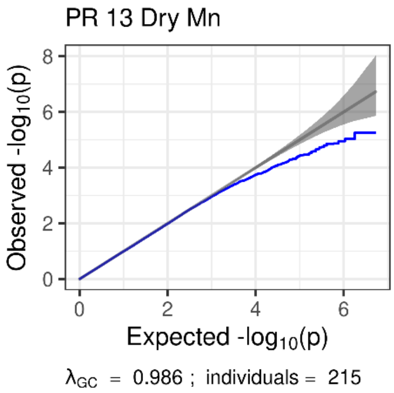

Molybdenum (Mo)

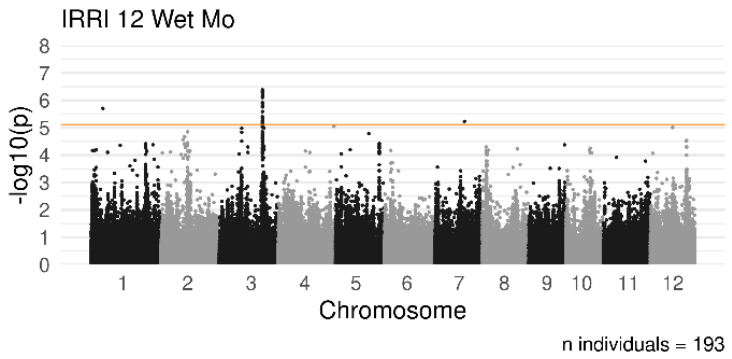

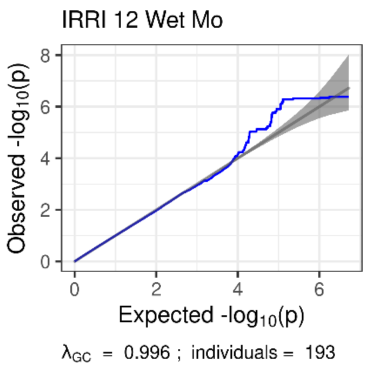

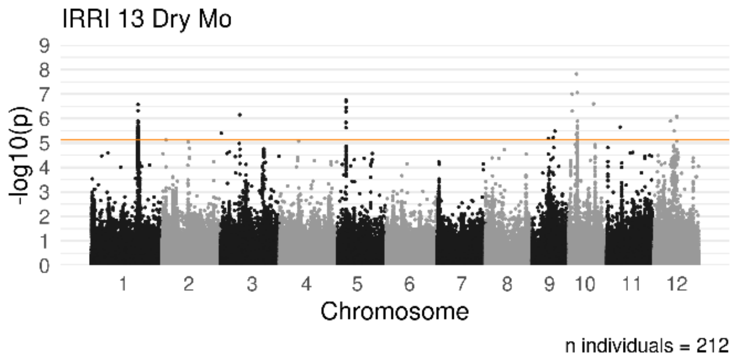

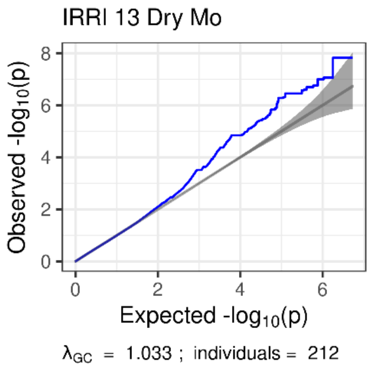

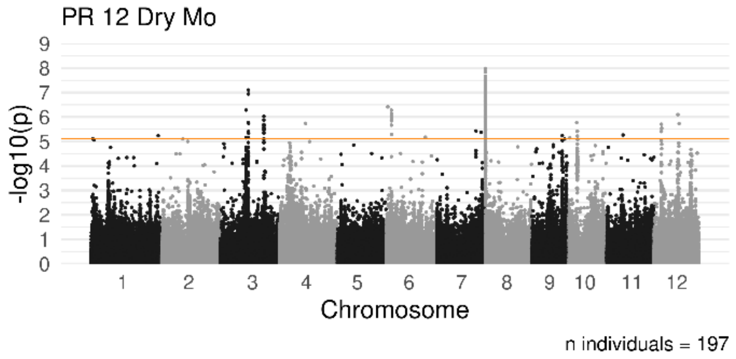

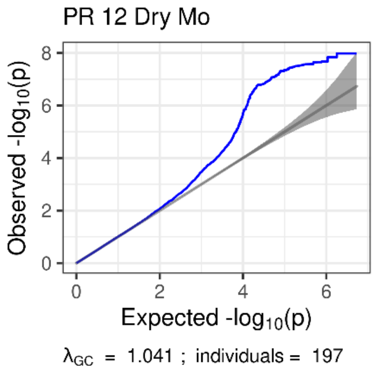

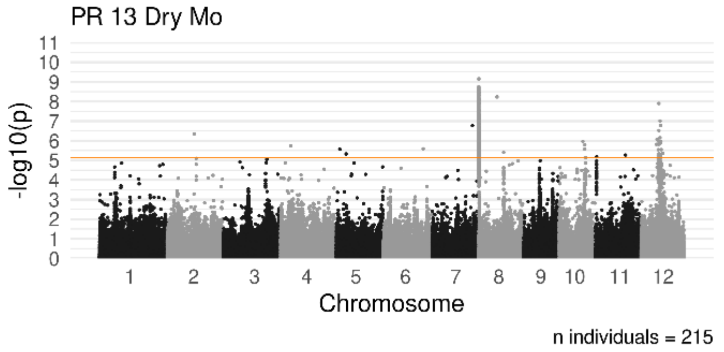

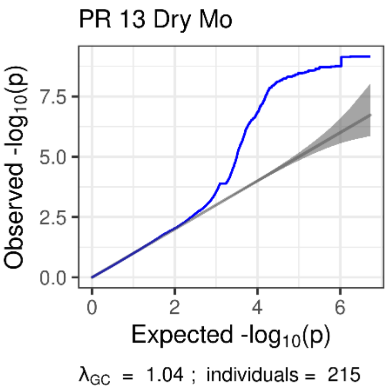

Sodium (Na)

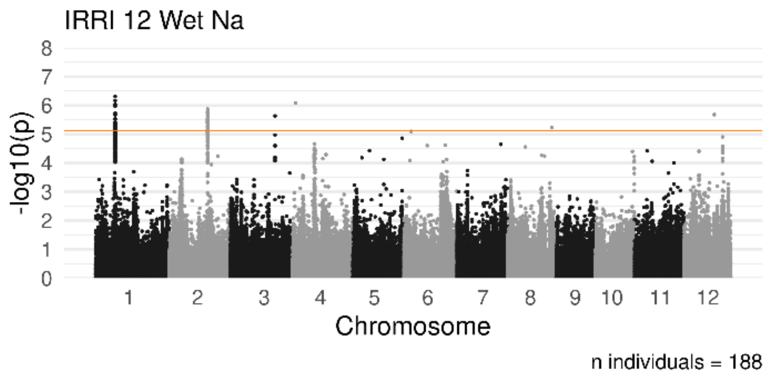
IRRI 12 Wet Na
-log10(p)
Chromosome
n individuals = 188

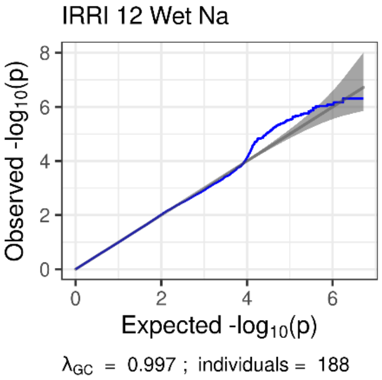
IRRI 12 Wet Na
Observed -log10(p)
Expected -log10(p)
λGC = 0.997 ; individuals = 188

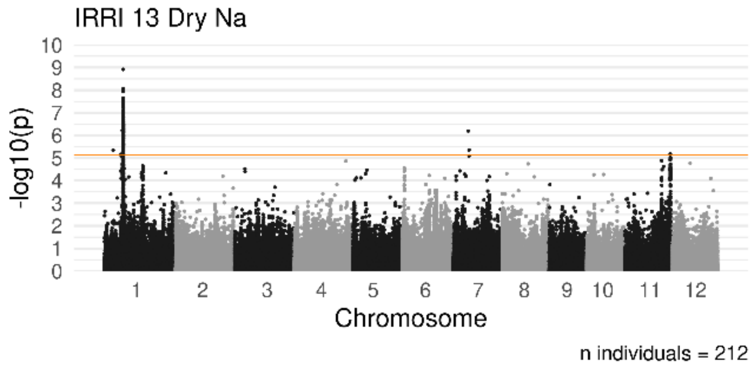
IRRI 13 Dry Na
-log10(p)
Chromosome
n individuals = 212

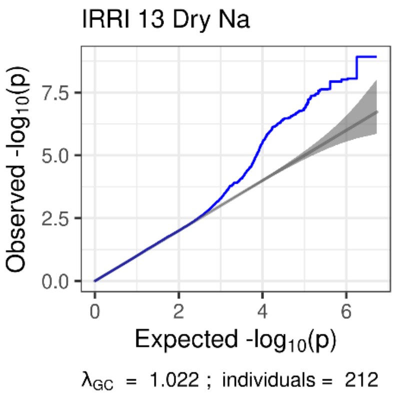
IRRI 13 Dry Na
Observed -log10(p)
Expected -log10(p)
λGC = 1.022 ; individuals = 212

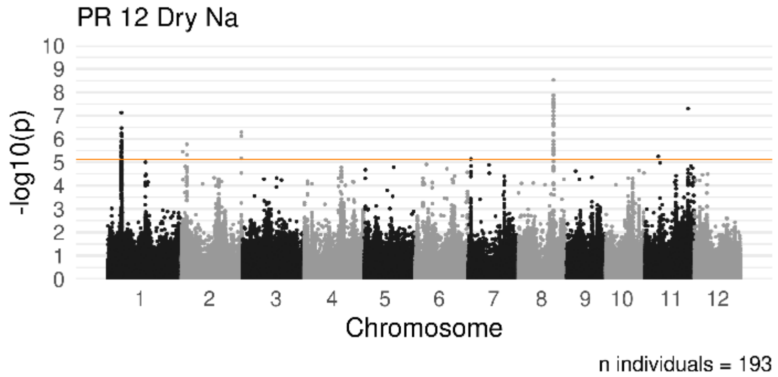
PR 12 Dry Na
-log10(p)
Chromosome
n individuals = 193

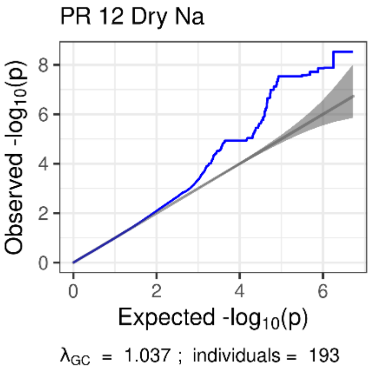
PR 12 Dry Na
Observed -log10(p)
Expected -log10(p)
λGC = 1.037 ; individuals = 193

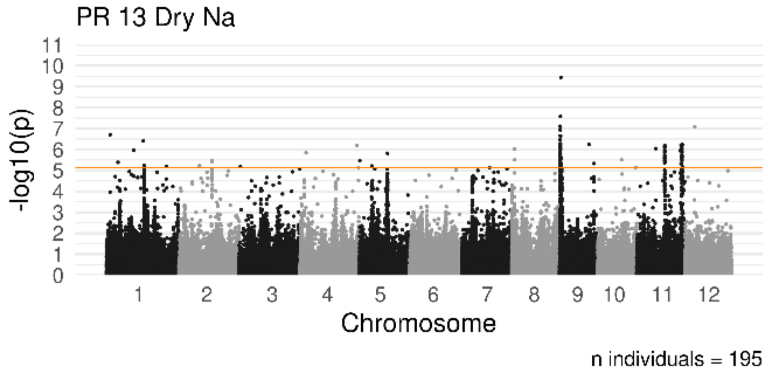
PR 13 Dry Na
-log10(p)
Chromosome
n individuals = 195

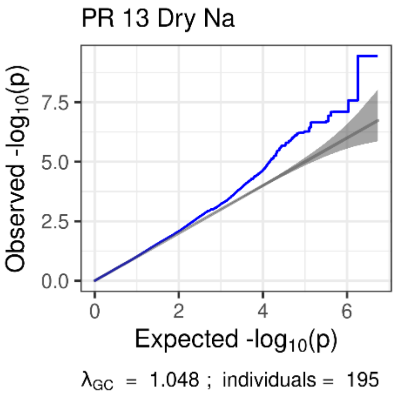
PR 13 Dry Na
Observed -log10(p)
Expected -log10(p)
λGC = 1.048 ; individuals = 195

Phosphorus (P)

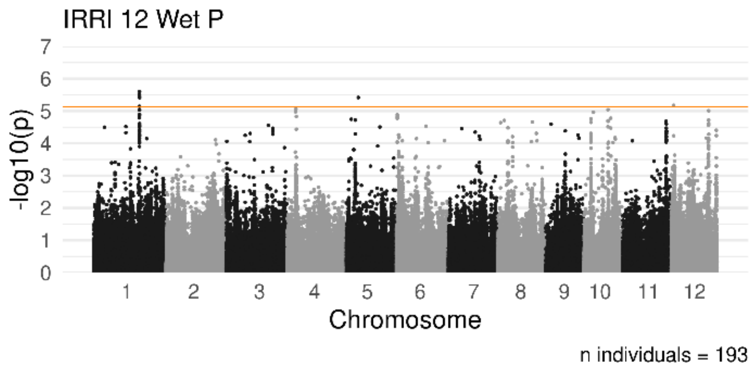

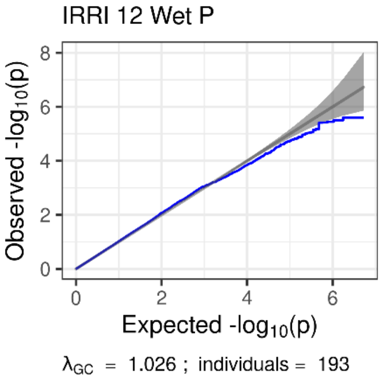

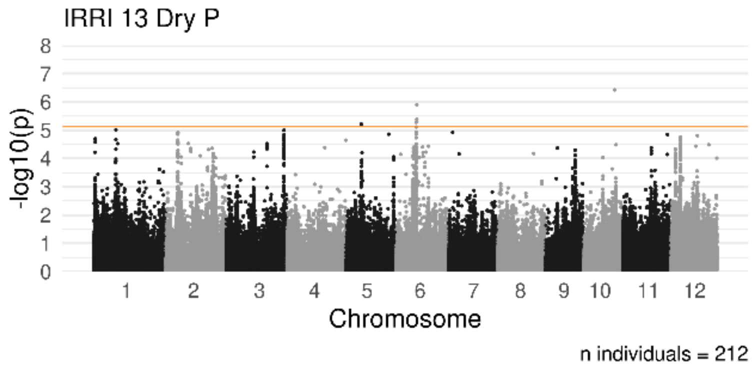

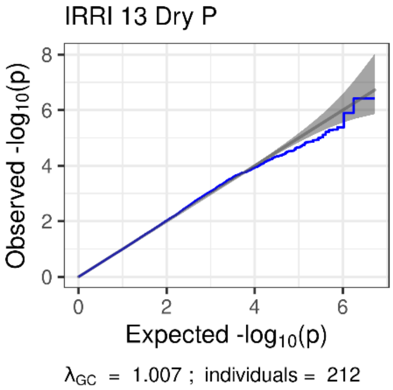

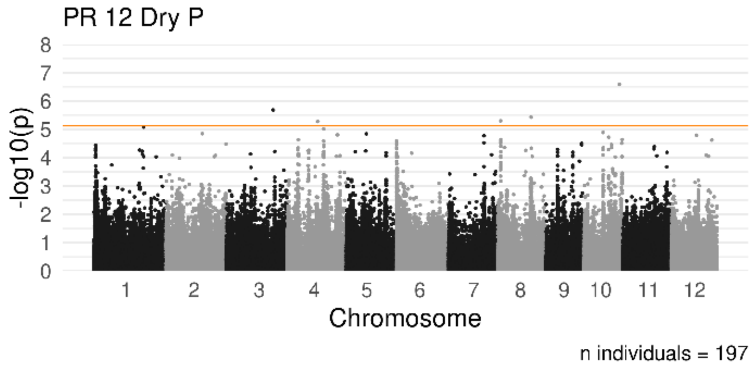

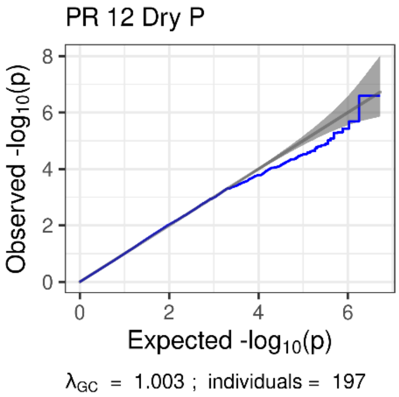

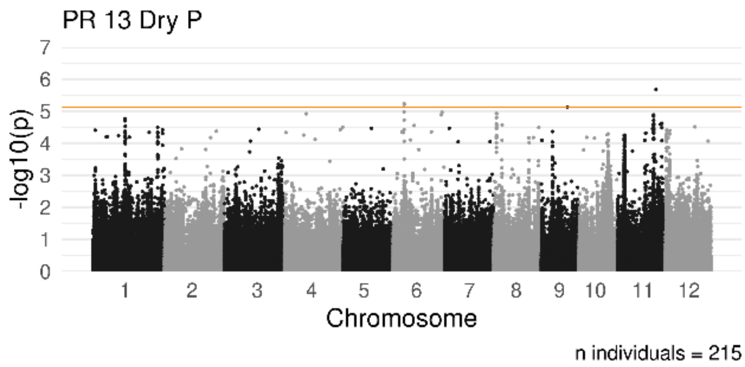

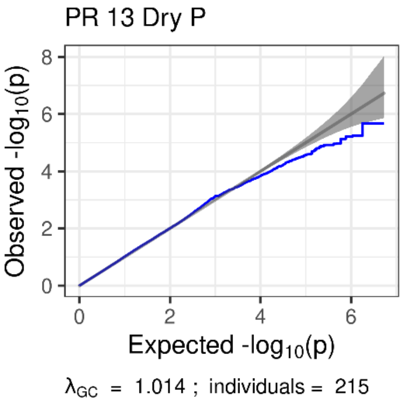

Days to flowering (DTFLW)

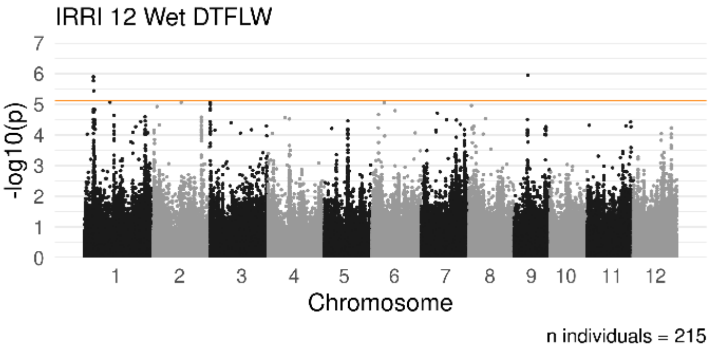

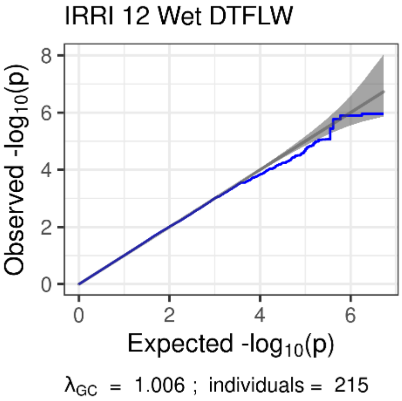

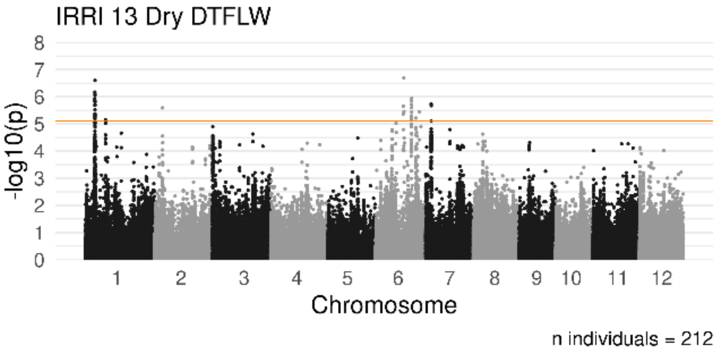

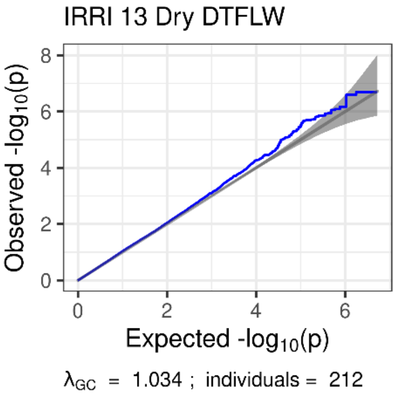

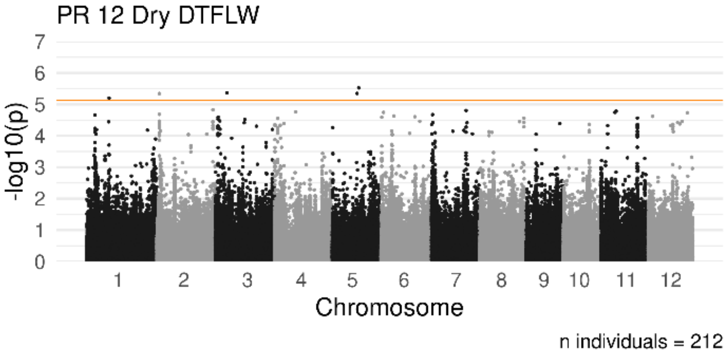

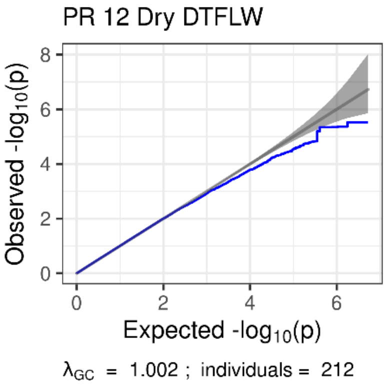

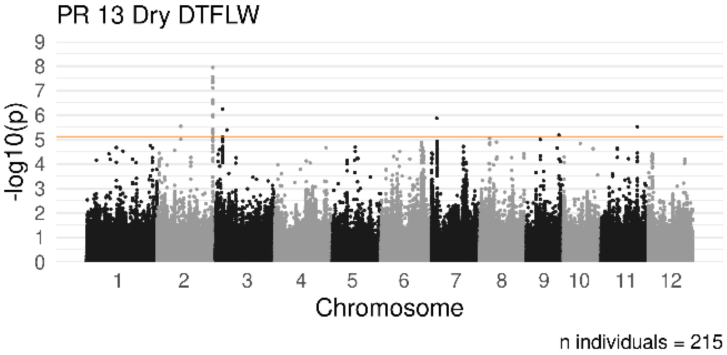

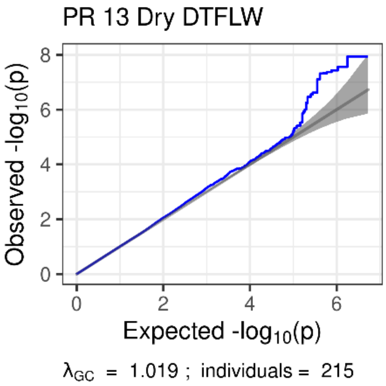

Grain yield (GY)

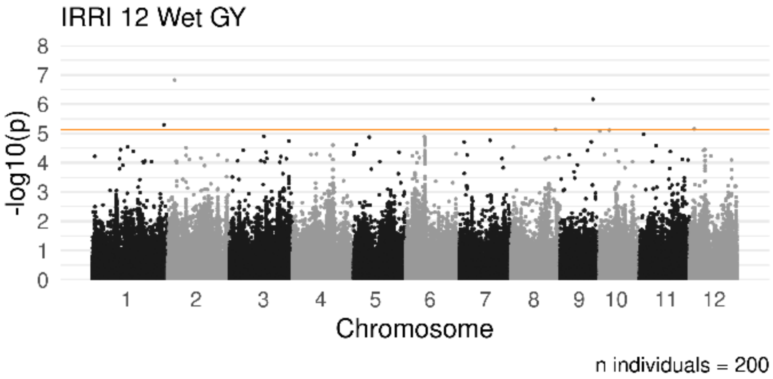

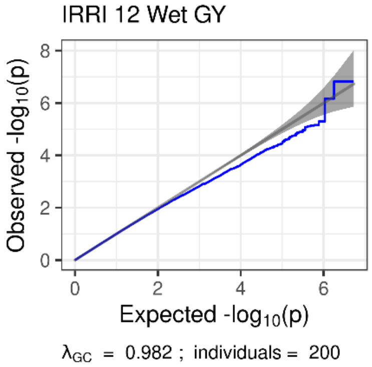

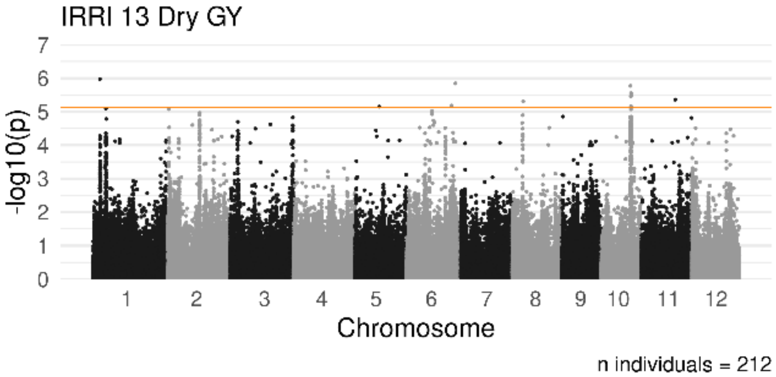

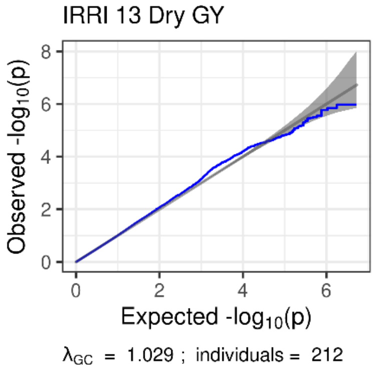

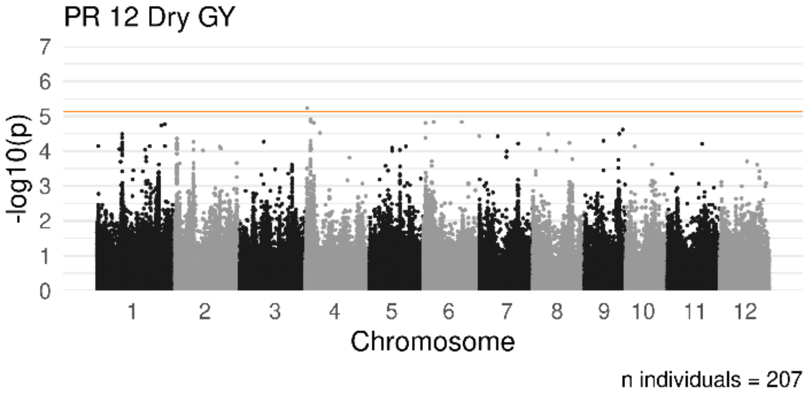

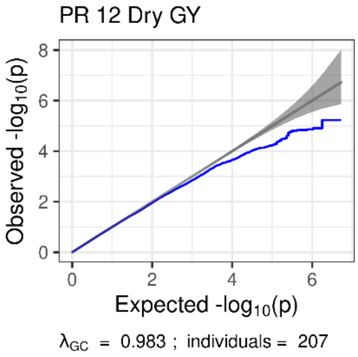

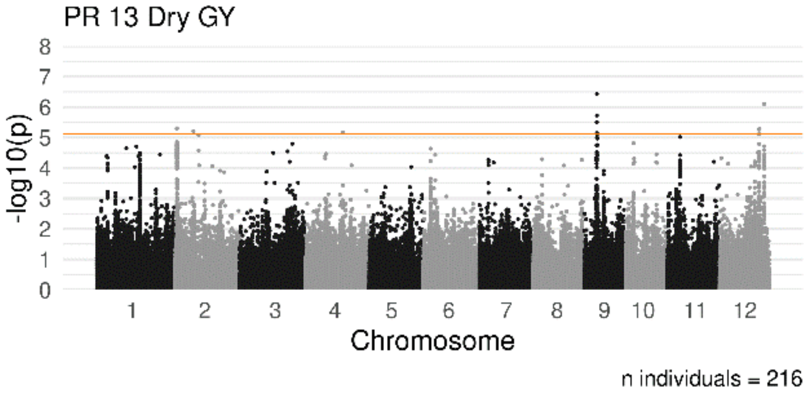

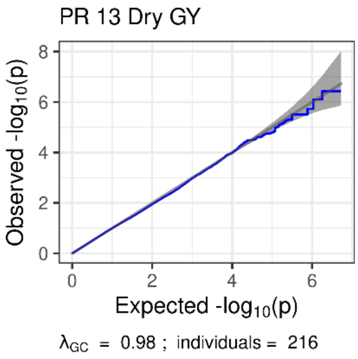

Plant height (PLTHT)

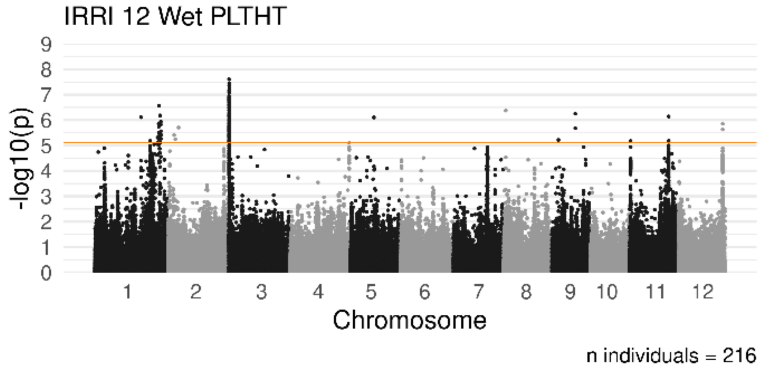

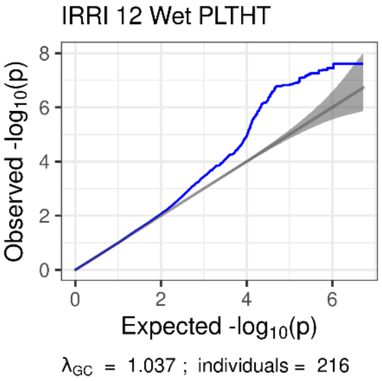

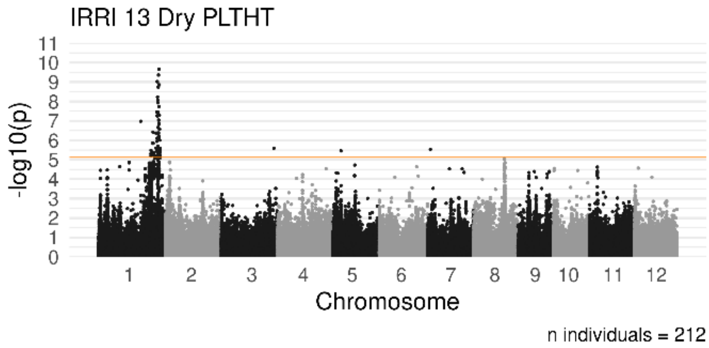

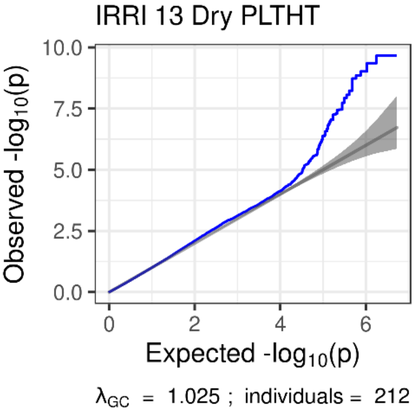

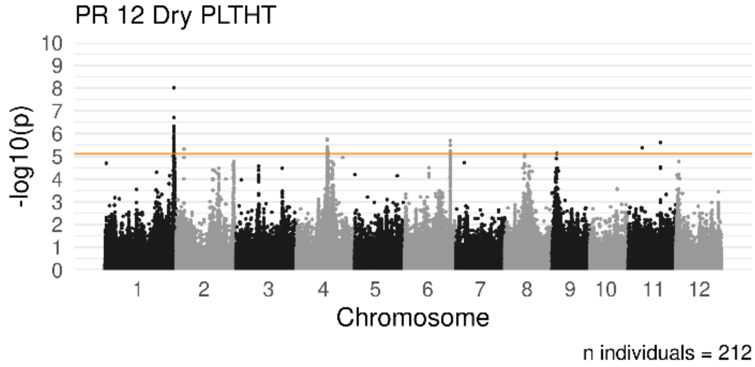

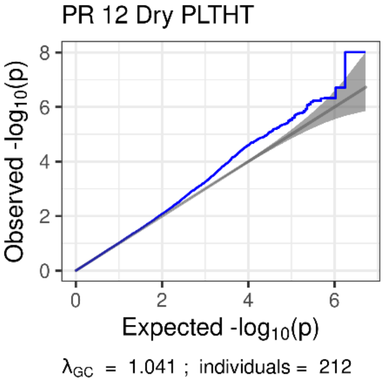

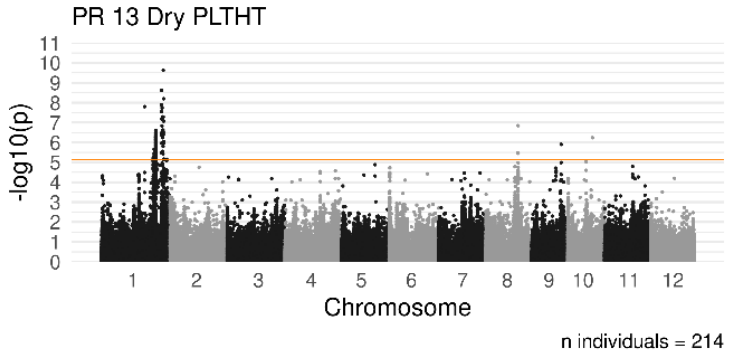

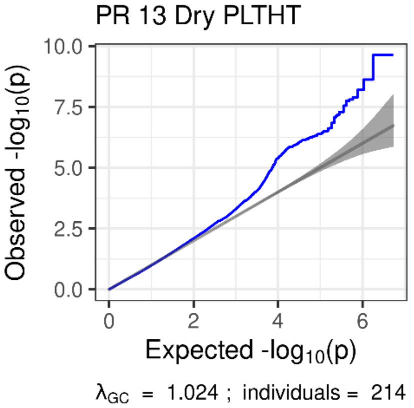

Thousand grain weight (TGWg)

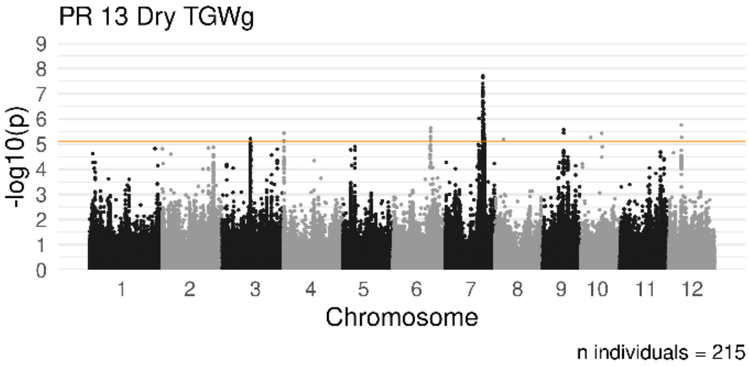

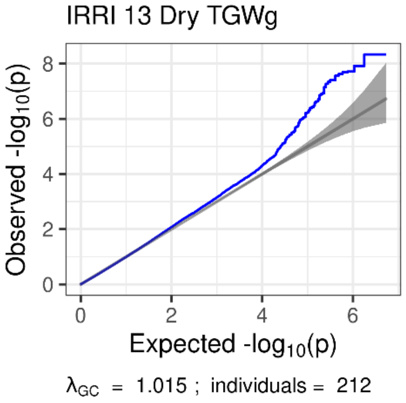

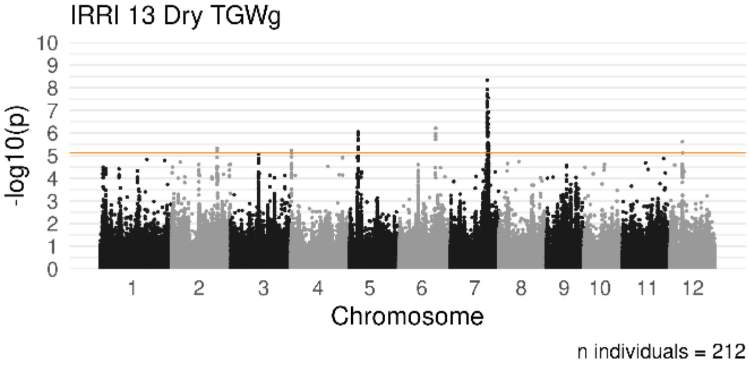

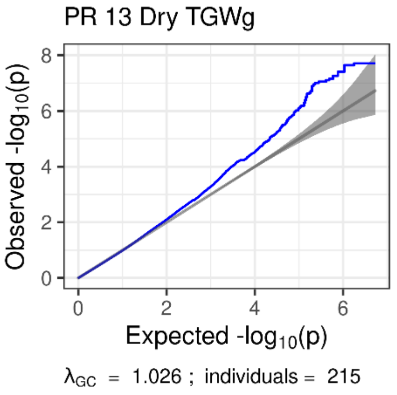

Supplement: Supplementary file 1 — Supplementary Information. [file 41598_2021_98573_MOESM1_ESM.pdf]
